# The MGF300-2R protein of African swine fever virus is associated with viral pathogenicity by promoting the autophagic degradation of IKK*α* and IKK*β* through the recruitment of TOLLIP

**DOI:** 10.1371/journal.ppat.1011580

**Published:** 2023-08-11

**Authors:** Tao Wang, Rui Luo, Jing Zhang, Zhanhao Lu, Lian-Feng Li, Yong-Hui Zheng, Li Pan, Jing Lan, Huanjie Zhai, Shujian Huang, Yuan Sun, Hua-Ji Qiu

**Affiliations:** 1 State Key Laboratory for Animal Disease Control and Prevention, National African Swine Fever Para-reference Laboratory, National High Containment Facilities for Animal Diseases Control and Prevention, Harbin Veterinary Research Institute, Chinese Academy of Agricultural Sciences, Harbin, China; 2 School of Life Science Engineering, Foshan University, Foshan, China; 3 Department of Microbiology and Molecular Genetics, Michigan State University, East Lansing, Michigan, United States of America; Pirbright Institute, UNITED KINGDOM

## Abstract

The multigene family genes (MGFs) in the left variable region (LVR) of the African swine fever virus (ASFV) genome have been reported to be involved in viral replication in primary porcine alveolar macrophages (PAMs) and virulence in pigs. However, the exact functions of key *MGFs* in the LVR that regulate the replication and virulence of ASFV remain unclear. In this study, we identified the *MGF300-2R* gene to be critical for viral replication in PAMs by deleting different sets of *MGFs* in the LVR from the highly virulent strain ASFV HLJ/18 (ASFV-WT). The ASFV mutant lacking the *MGF300-2R* gene (Del2R) showed a 1-log reduction in viral titer, and induced higher IL-1*β* and TNF-*α* production in PAMs than did ASFV-WT. Mechanistically, the MGF300-2R protein was found to interact with and degrade IKK*α* and IKK*β* via the selective autophagy pathway. Furthermore, we showed that MGF300-2R promoted the K27-linked polyubiquitination of IKK*α* and IKK*β*, which subsequently served as a recognition signal for the cargo receptor TOLLIP-mediated selective autophagic degradation. Importantly, Del2R exhibited a significant reduction in both replication and virulence compared with ASFV-WT in pigs, likely due to the increased IL-1*β* and TNF-*α*, indicating that MGF300-2R is a virulence determinant. These findings reveal that MGF300-2R suppresses host innate immune responses by mediating the degradation of IKK*α* and IKK*β*, which provides clues to paving the way for the rational design of live attenuated vaccines to control ASF.

## Author summary

The left variable region in the genome of African swine fever virus (ASFV) contains a large number of multigene family genes (MGFs), but the impact of individual *MGFs* on viral replication and pathogenicity is largely unknown. In this study, we identified MGF300-2R as a new virulence factor, which is critical for viral replication *in vivo* and *in vitro*. Furthermore, we showed that MGF300-2R promotes the K27-linked ubiquitination of IKK*α* and IKK*β* for TOLLIP-dependent autophagic degradation, serving as a suppressor to prevent the activation of the NF-*κ*B signaling pathway. Our findings highlight the relevance of the inflammatory response in the virulence of ASFV and imply MGF300-2R as a candidate target for the rational design of vaccines against ASFV infections.

## Introduction

African swine fever virus (ASFV) is a large, double-stranded DNA virus that belongs to the genus *Asfivirus* in the family *Asfarviridae*. ASFV is the causal agent of African swine fever (ASF), a hemorrhagic and often lethal disease of domestic pigs and wild boars [1]. ASF has become endemic or epidemic in nearly 50 countries in Africa, Europe, Asia, and the Caribbean, causing huge economic losses that are difficult to estimate and posing a critical threat to the global pig industry and related industries [1–3]. Currently, only one ASF live attenuated vaccine (LAV) was marketed in Vietnam, but its use is restricted [4]. Importantly, previous experimental studies have revealed that LAV candidates have shown higher levels of protection than other types of ASF vaccines [5]. Hence, it is essential to discover and characterize the virulence-associated genes of ASFV for effective control ASF and future development of strategies for LAVs [5].

The ASFV genome varies in length between approximately 170 and 194 kilobase-pairs (kb), mainly due to variable copies of the multigene family genes (MGFs) from different isolates [6–8]. *MGFs* are mainly located at both ends of the ASFV genome and are referred to as left and right variable regions (LVR and RVR), which are divided into five clusters (MGF100, MGF110, MGF300, MGF360, and MGF505) based on the average amino acid lengths. The *MGFs* encode proteins that have been reported to be associated with host antiviral immunity modulation, viral virulence, cell tropism, and host range [9,10]. ASFV virulence has been associated with MGF360 and MGF505 genes which are non-essential for virus replication in primary porcine alveolar macrophages (PAMs). The deletion of six genes of MGF360 (*12L*, *13L*, and *14L*) and MGF505 (*1R*, *2R*, and *3R*) together led to decreased virulence in pigs, but the individual contribution of each *MGF* to attenuation remains unknown [11]. Recent studies have shown that the MGF505-7R protein can antagonize the production of interferon (IFN) and inflammatory cytokines in PAMs and pigs [12]. More importantly, the deletion of the *MGF505-7R* gene from a virulent strain partially attenuated its virulence in pigs [13–16]. The pandemic of ASF highlights the importance of studying the molecular mechanism of individual *MGFs* involved in pathogenesis, which is still largely unknown [17,18].

In mammals, the nuclear factor kappa B (NF-*κ*B) family comprises a group of related transcription factors that control the expression of hundreds of genes associated with diverse cellular host responses, such as inflammation, programmed cell death, cell growth, and survival [19]. In most cells, the NF-*κ*B complex remains inactive predominantly in the cytoplasm through binding to the inhibitor kappa-B alpha (I*κ*B*α*). Toll-like receptors (TLRs) and IL-1 receptors transmit signals to the I*κ*B kinase (IKK) complex, which consists of NEMO and two kinases, IKK*α* and IKK*β*, via IRAK1 and TRAF6 [20]. In the canonical NF-*κ*B pathway, various stimuli trigger the phosphorylation of I*κ*B*α* via IKK*β*, leading to I*κ*B*α* ubiquitination and subsequent proteasomal degradation [21]. The released NF-*κ*B-p65 is translocated to the nucleus, binds *κ*B-responsive DNA sequences, recruits transcription co-regulators, and modulates hundreds of gene expressions. NF-*κ*B plays a critical role in many cellular responses, and it is not surprising that most viruses have evolved strategies to subvert the NF-*κ*B signaling pathway [20,21]. For example, vaccinia virus (VACV) encodes at least eleven NF-*κ*B antagonists, including A46R, A49, A52, B14, C4, F14, E3, K1L, K7, M2L, and N1L, and can inhibit the NF-*κ*B activation at different levels [21–23]. Similarly, ASFV encodes several proteins, including H240R, A238L, F317L, MGF505-7R, and D345L that can inhibit the production of interleukin 1beta (IL-1*β*) and IFNs by modulating NF-*κ*B activation [24–28]. However, the exact impact of these proteins on the pathogenesis of ASFV is still not fully understood.

Autophagy is an evolutionarily conserved mechanism cellular process that plays a crucial role in maintaining cellular homeostasis and clearance of invading pathogens [29]. Selective autophagy targets specific substrates mediated by selective autophagy receptors (SARs), such as p62/SQSTM1, TOLLIP, NDP52, OPTN, NBR1, and TAX1BP1 [30]. These SARs recognize the ubiquitinated substrate proteins and bind to components of the core autophagic machinery, such as microtubule-associated protein 1 light chain 3 (LC3), Atg11 (in yeasts), or FIP200 (in mammals), to regulate protein degradation [30]. In addition, SARs have been shown to suppress viral infection *in vitro*, including herpes simplex virus type 1 (HSV1), Zika virus (ZIKV), Ebola virus (EBOV), and porcine epidemic diarrhea virus (PEDV) by degrading the viral proteins [31–33]. However, the interaction between viral proteins and SARs during ASFV infection is still poorly understood.

We demonstrated previously that the cell-adapted ASFV with a 24.5-kb genes deletion in the LVR lost the ability to replicate in PAMs [34]. Recently, we found that the deleted LVR genes of the cell-adapted ASFV may contain critical *MGFs* for ASFV replication in PAMs [35]. In this study, we reveal that the MGF300 genes (*1L*, *2R*, and *4L*) encoding proteins are critical for the replication of ASFV in PAMs. Additionally, we have demonstrated that MGF300-2R functions as an inhibitor of the NF-*κ*B signaling pathway by specifically targeting IKK*α* and IKK*β* for autophagic degradation, thereby preventing the production of IL-1*β* and TNF-*α in vitro* and *in vivo*. Deletion of the *MGF300-2R* gene resulted in partial attenuation of the ASFV in pigs, as evidenced by reduced viral replication and increased production of IL-1*β* and TNF-*α*. Our findings demonstrate an important mechanism of MGF300-2R hijacks the NF-*κ*B signaling pathway to facilitate ASFV pathogenesis.

## Results

### The *MGF300* genes (*1L*, *2R*, and *4L*) are critical to ASFV replication in PAMs

**Fig 1:**
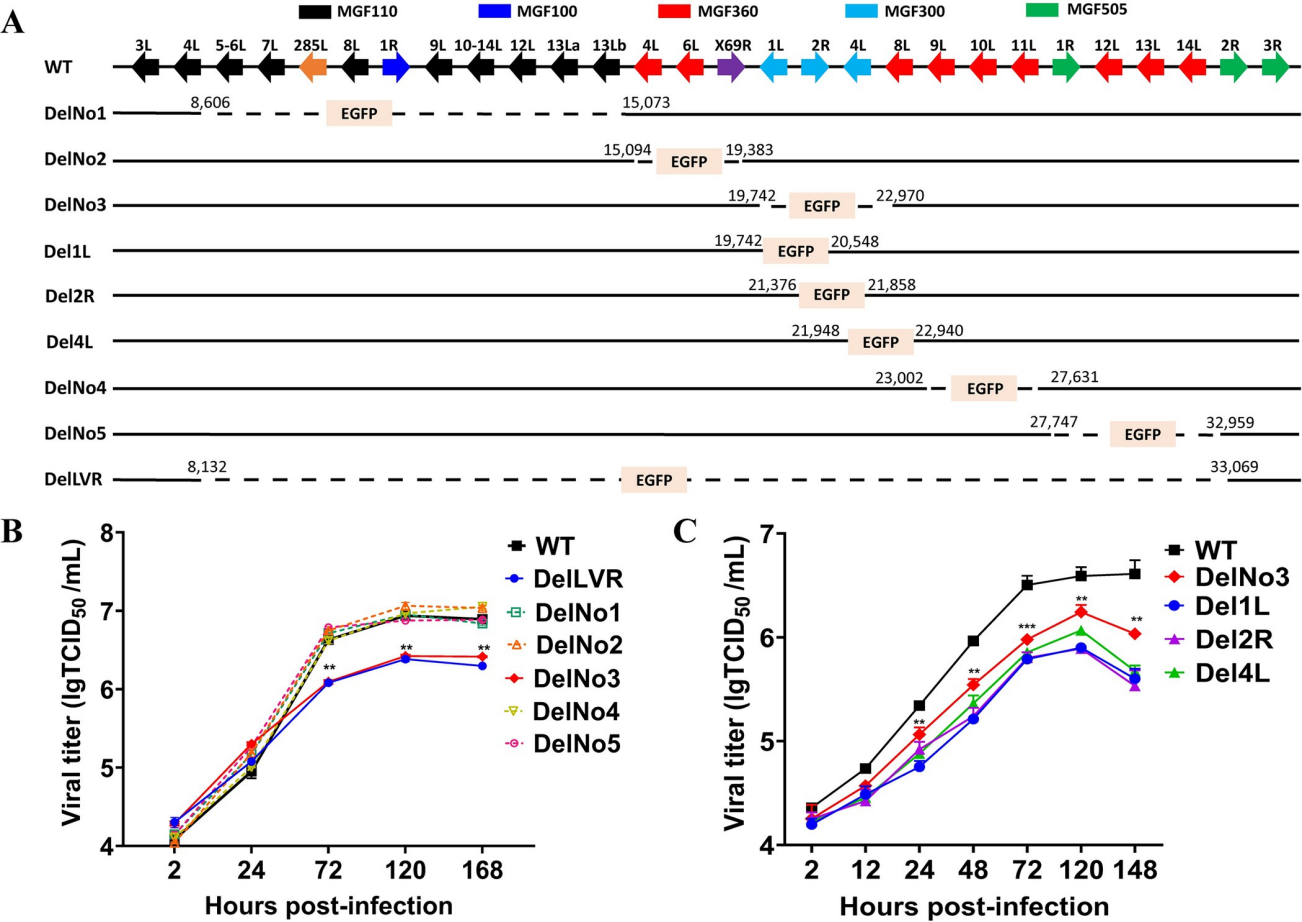
*MGF300* genes are critical to ASFV replication in primary porcine alveolar macrophages (PAMs). (A) Schematic diagram depicting the deletion of multigene family genes (MGFs) in the left variable region (LVR) of the ASFV HLJ/18 strain (ASFV-WT) genome. Dashed lines indicate the boundaries of the deleted *MGFs* relative to the ASFV-WT genome are indicated. (B and C) *In vitro* growth characteristics of ASFV-WT and the mutants with different deletion combinations of *MGFs*. PAMs were infected (MOI = 5) with each of the viruses and the virus yield was titrated at the indicated times post-infection.

To identify the individual *MGFs* deleted from ASFV-WT that are critical to ASFV replication in PAMs, we generated six ASFV mutants with different combinations of *MGFs* deletion in LVR using homologous recombination (**Fig 1A**). The *MGFs*-deleted recombinant ASFVs were purified via successive limited dilution by selecting EGFP-positive PAMs. The purity of these *MGFs*-deleted recombinant ASFVs was confirmed by PCR (**S1A Fig**). Furthermore, the whole-genome sequence of DelLVR was determined by NGS as described previously [35]. Next, the replication kinetics of the *MGFs*-deleted recombinant ASFVs were compared with that of ASFV-WT in PAMs, and it was found that ASFV mutants DelNo1, DelNo2, DelNo4, and DelNo5 exhibited similar growth kinetics to ASFV-WT. However, lower replication levels were observed for DelNo3 and DelLVR between 3 to 7 dpi compared with ASFV-WT (**Fig 1B**). These findings suggested that the deletion of the *MGF300* genes (*1L*, *2R*, and *4L*) significantly decreased viral replication, indicating that these genes are critical for viral replication in PAMs.

To further identify the role of the individual viral genes of MGF300 that regulate viral replication in PAMs, we constructed three recombinant viruses with single deletion of the *MGF300-1L* (Del1L), *MGF300-2R* (Del2R), or *MGF300-4L* (Del4L) gene. The whole-genome sequencing and analysis of the Del1L, Del2R, and Del4L confirmed that all the final recombinant viruses harbored the correct sequences (**S1B–S1D Fig** and **S2 Table**). The replication kinetics of Del1L, Del2R, and Del4L were similar but significantly lower than that of ASFV-WT after 3 dpi (**Fig 1C**). These data confirm that the *MGF300* genes are critical to ASFV replication in PAMs. In this study, we proceed to further investigate MGF300-2R as it is a highly conserved protein localized in the cytoplasm and nucleus with nuclear localization signal (**S2A–S2D Fig**), which may be important for ASFV infection.

### The *MGF300-2R*-deleted ASFV mutant induces higher production of inflammatory cytokines than does the wild-type ASFV in PAMs

To investigate the role of MGF300-2R protein in the ASFV life cycle, we compared the binding, internalization, and the release of virions of Del2R and ASFV-WT, but no significant differences were observed between the two viruses (**S3A and S3B Fig**). As MGF300-2R is highly conserved and localized in both the cytoplasm and nucleus (**S2C Fig.**), it may play a role in ASFV morphogenesis as a structural protein. However, TEM analysis revealed no differences in the ultrastructure of viral factories or virion morphogenesis between Del2R and ASFV-WT (**S3C and S3D Fig**). Therefore, we conclude that MGF300-2R is not essential for ASFV replication in PAMs.

**Fig 2:**
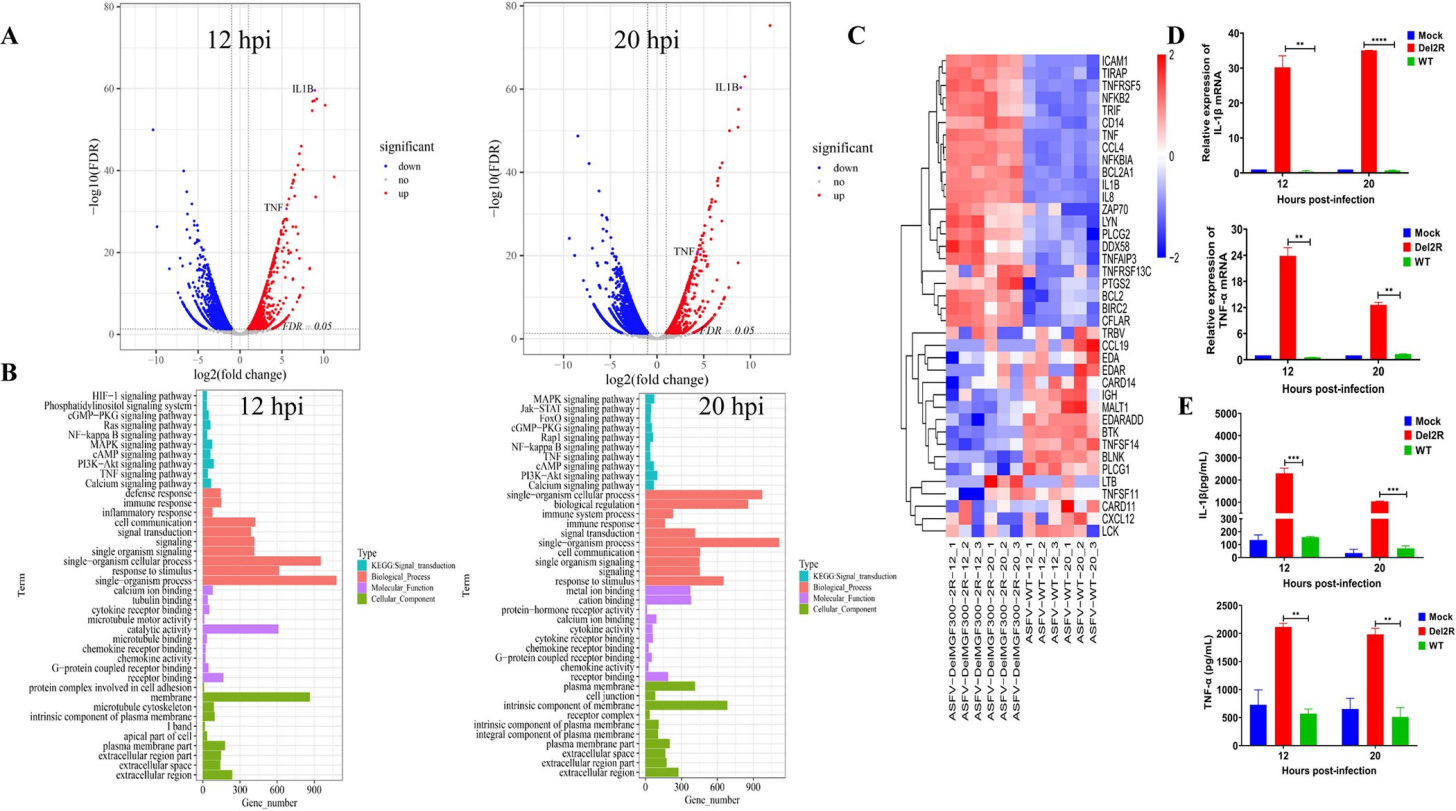
The MGF300-2R-deleted ASFV mutant (Del2R) induces higher production of inflammatory cytokines than does the wild-type ASFV (ASFV-WT) in PAMs. (A) PAMs were mock infected or infected with Del2R or ASFV-WT (MOI = 5) for 12 and 20 h for RNA-seq analysis. Volcano plot of gene changes in Del2R-infected PAMs compared with the expression in ASFV-WT-infected PAMs. Red dots and blue dots denote upregulated or downregulated differentially expressed genes (DEGs), respectively. (B) Gene ontology (GO) category functional enrichment by 3 categories (Biological process, Molecular function, and Cell component) and Kyoto encyclopedia of genes and genomes (KEGG) pathway analysis was performed for up-regulated genes in the group of Del2R versus ASFV-WT. (C) The heatmaps show the expression levels of DEGs in the NF-*κ*B signaling pathway induced by Del2R versus ASFV-WT at 12 and 20 hours post-infection (hpi). (D and E) PAMs were either mock infected or infected with Del2R or ASFV-WT (MOI = 5). At 12 and 20 hpi, the mRNA levels of IL-1*β* and TNF-*α* (D) in the cell lysates were determined by RT-qPCR, and the production of IL-1*β* and TNF-*α* (E) in the cell culture supernatants were detected by commercial ELISA kits.

Next, to further dissect the role of MGF300-2R in ASFV replication, RNA-seq analysis was used to investigate the host immune responses induced by infection with Del2R in PAMs. Our results revealed a total of 4159 differentially expressed genes (DEGs) for Del2R vs. ASFV-WT at 12 hpi **(S3 Table)**, including 1326 upregulated and 2833 downregulated genes, and a total of 4525 DEGs for Del2R vs. ASFV-WT at 20 hpi **(S3 Table)**, including 1007 upregulated and 3518 downregulated genes (**Fig 2A**). It is of significance to observe that, in comparison with ASFV-WT, the expression level of IL-1*β* was markedly increased by approximately 9-fold at 12 and 20 hpi (**Fig 2A**). In comparison to PAMs infected with ASFV-WT, the differentially expressed mRNAs during Del2R infection were mainly involved in innate immunity signaling pathways such as NF-*κ*B, TNF, MAPK, and PI3K-AKT signaling pathways, as well as receptor signaling, metabolic networks, and other pathways associated with viral infection (**Fig 2B**). The altered cellular mRNAs from NF-*κ*B signaling pathways are presented in **Fig 2C**, wherein IL-1*β*, TNF-*α*, and IL-6 were found to be highly transcribed during Del2R infection compared with ASFV-WT at 12, and 20 hpi. To further validate the RNA-seq results, the levels of cytokines IL-1*β*, TNF-*α*, and IFN-*α* were measured by RT-qPCR. Del2R infection significantly increased the expression of IL-1*β* and TNF-*α* mRNA by approximately 20-fold at 12 and 20 hpi, compared with ASFV-WT (**Fig 2D**). Likewise, the secretion of IL-1*β* and TNF-*α* induced by Del2R was approximately 20-fold higher than that induced by ASFV-WT at 12 and 20 hpi in PAMs (**Fig 2E**). However, no differences in IFN-*α* expression level was observed between the two viruses (**S4A and S4B Fig**). Altogether, these findings suggest that Del2R activates the inflammatory signaling pathway in PAMs.

### MGF300-2R interacts with IKK*α* and IKK*β* and inhibits the NF-*κ*B activation

**Fig 3:**
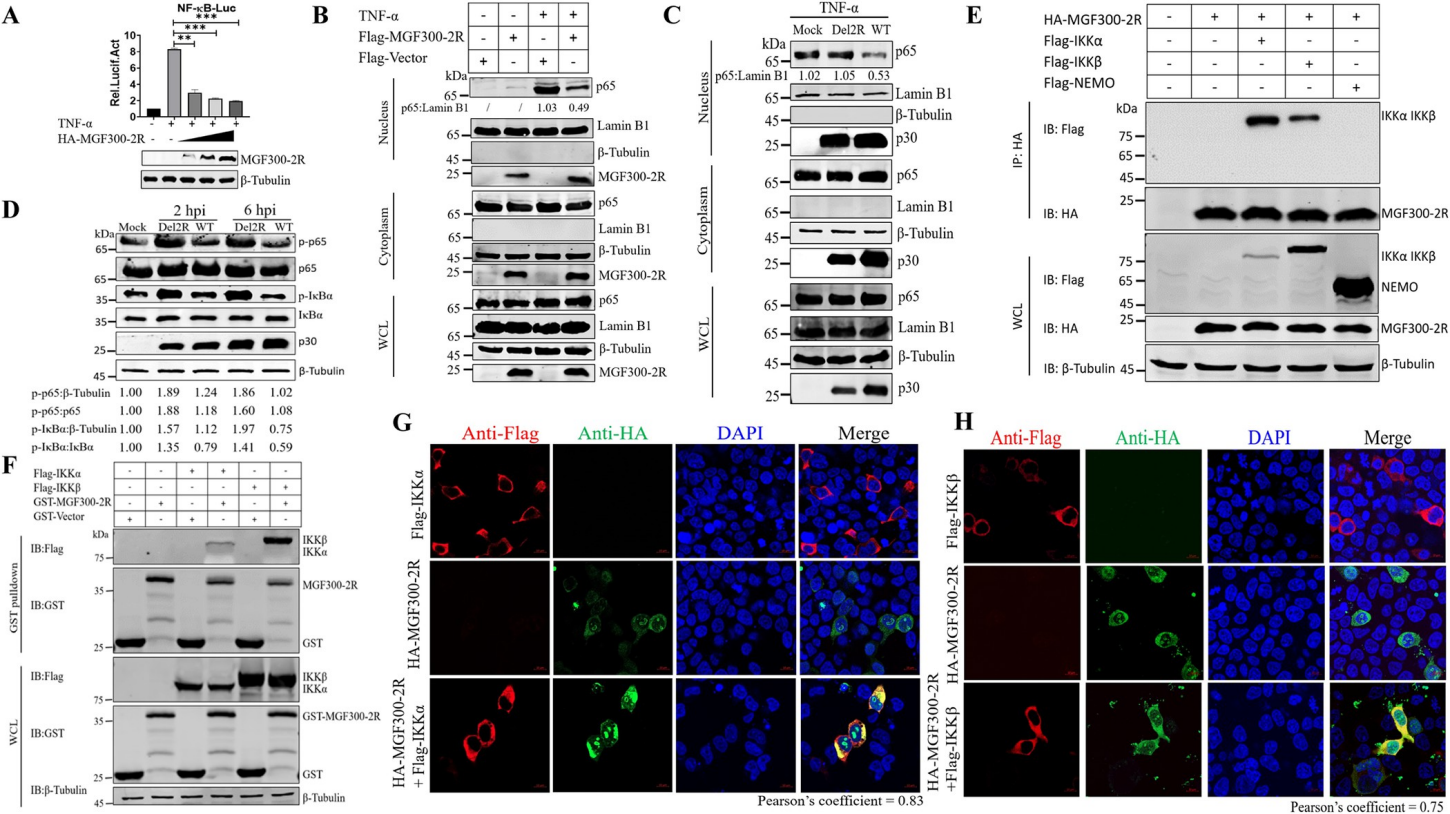
The MGF300-2R protein interacts with IKK*α* and IKK*β* and inhibits the NF-*κ*B activation. (A) HEK293T cells were transfected with increasing amounts of the expressing plasmid pHA-MGF300-2R (0.5, 1, and 1.5 *μ*g) for 24 h and then mock-treated or treated with TNF-*α* (10 ng/mL) for 8 h. The protein expression of MGF300-2R was examined by western blotting and the luciferase activity was measured at 24 hours post-transfection (hpt). (B) HEK293T cells were transfected with the pFlag-MGF300-2R or empty plasmid and then treated with or without TNF-*α* (10 ng/mL) for 1 h. At 24 hpt, the cells were fractionated into cytoplasmic and nuclear fractions and analyzed by immunoblotting with the indicated antibodies. The p65 in the nuclear and cytoplasmic compartments was checked by western blotting. Lamin B1 and tubulin were used as nuclear and cytosolic markers, respectively. (C) PAMs were mock-infected or infected with ASFV-WT or Del2R (MOI = 5). At 20 hpi, PAMs were treated with or without TNF-*α* (10 ng/mL) for 1 h, the separation of cellular fractions of ASFV-infected PAMs was performed as described above. (D) Analysis of the phosphorylation levels of I*κ*B*α* and p65 in PAMs mock-infected or infected with ASFV-WT, or Del2R (MOI = 5) by western blotting at 2 and 6 hpi. (E) HEK293T cells were transfected with a plasmid encoding HA-MGF300-2R along with a plasmid encoding Flag-IKK*α*, Flag-IKK*β*, or Flag-NEMO as indicated. The cells were lysed and whole cell lysates (WCL) were immunoprecipitated with anti-HA mAb at 36 hpt. The immunoprecipitates were examined by western blotting with the indicated antibodies. (F) HEK293T cells were transfected with expressing plasmids pFlag-IKK*α* or pFlag-IKK*β* for 36 h and lysed with NP-40 buffer. The purified GST or GST-MGF300-2R protein was used to pull down the IKK*α* or IKK*β* in the lysates and analyzed by western blotting with the indicated antibodies. (G and H) HEK293T cells were transfected with pHA-MGF300-2R alone and cotransfected with pFlag-IKK*α* or pFlag-IKK*β* in combination with pHA-MGF300-2R. IKK*α*, IKK*β*, and MGF300-2R were analyzed by laser confocal microscopy. Scale bar, 10 *μ*m. The colocalization of MGF300-2R and IKK*α* or IKK*β* was analyzed by the Coloc2 tool of ImageJ/FIJI and shown as Pearson’s correlation coefficients.

It is well-known that ASFV has evolved various mechanisms to evade the host antiviral innate immune signaling and promote its replication and pathogenesis [36]. In light of this, we hypothesized that MGF300-2R may facilitate viral replication by counteracting the inflammatory signaling pathways mediated by the NF-*κ*B. To test this hypothesis, we conducted experiments to assess the effects of MGF300-2R on NF-*κ*B activation. We found that the ectopically expressed MGF300-2R inhibited the TNF-*α*-triggered activation of the NF-*κ*B promoter in a dose-dependent manner (**Fig 3A**), while not significantly affecting other reporters such as IFN-*β* (**S4C Fig**), IRF3 (**S4D Fig**), and ISRE (**S4E and S4F Fig**).

Additionally, subcellular fractionation experiments showed that the ectopically expressed MGF300-2R inhibited the nuclear translocation of p65 **(Fig 3B)**. Next, we investigated the nuclear localization pattern of p65 in the infected PAMs. As shown in **Fig 3C**, p65 in the nuclear fraction of the Del2R-infected PAMs was significantly increased compared with that of the ASFV-WT-infected PAMs. To further confirm the inhibitory effects of MGF300-2R on the NF-*κ*B signaling, the PAMs infected with Del2R or ASFV-WT were assessed for the phosphorylation of p65 and I*κ*B*α*, two hallmarks of NF-*κ*B activation. The results showed that infection with Del2R led to more phosphorylation of p65 and I*κ*B*α* than did ASFV-WT infection at 2 and 6 hpi in PAMs **(Fig 3D)**. Taken together, these results corroborate the conclusion that MGF300-2R negatively regulates the NF-*κ*B-mediated innate immune response.

We then tried to determine the potential mechanism(s) by which MGF300-2R inhibits the NF-*κ*B signaling pathway. Our results showed that MGF300-2R functions upstream of I*κ*B*α* by inhibiting the phosphorylation of p65 and I*κ*B*α*, and the production of IL-1*β* and TNF-*α*. To investigate whether MGF300-2R can interact with any protein in the IKK complex, we performed co-immunoprecipitation (co-IP) experiments in HEK293T cells. We found that MGF300-2R specifically co-precipitated with both IKK*α* and IKK*β*, but not NEMO **(Fig 3E)**. This interaction was further validated through a GST pulldown assay, in which GST-MGF300-2R pulled down Flag-tagged IKK*α* and IKK*β* **(Fig 3F)**. Confocal microscopy analysis confirmed that MGF300-2R and IKK*α* or IKK*β* were colocalized in the cytoplasm of HEK293T cells **(Fig 3G and 3H)**. Taken together, these results showed that MGF300-2R interferes with the IKK complex to counteract the activation of the NF-*κ*B signaling pathway.

### Autophagic degradation of IKK*α* and IKK*β* can be induced by the ASFV MGF300-2R protein

**Fig 4:**
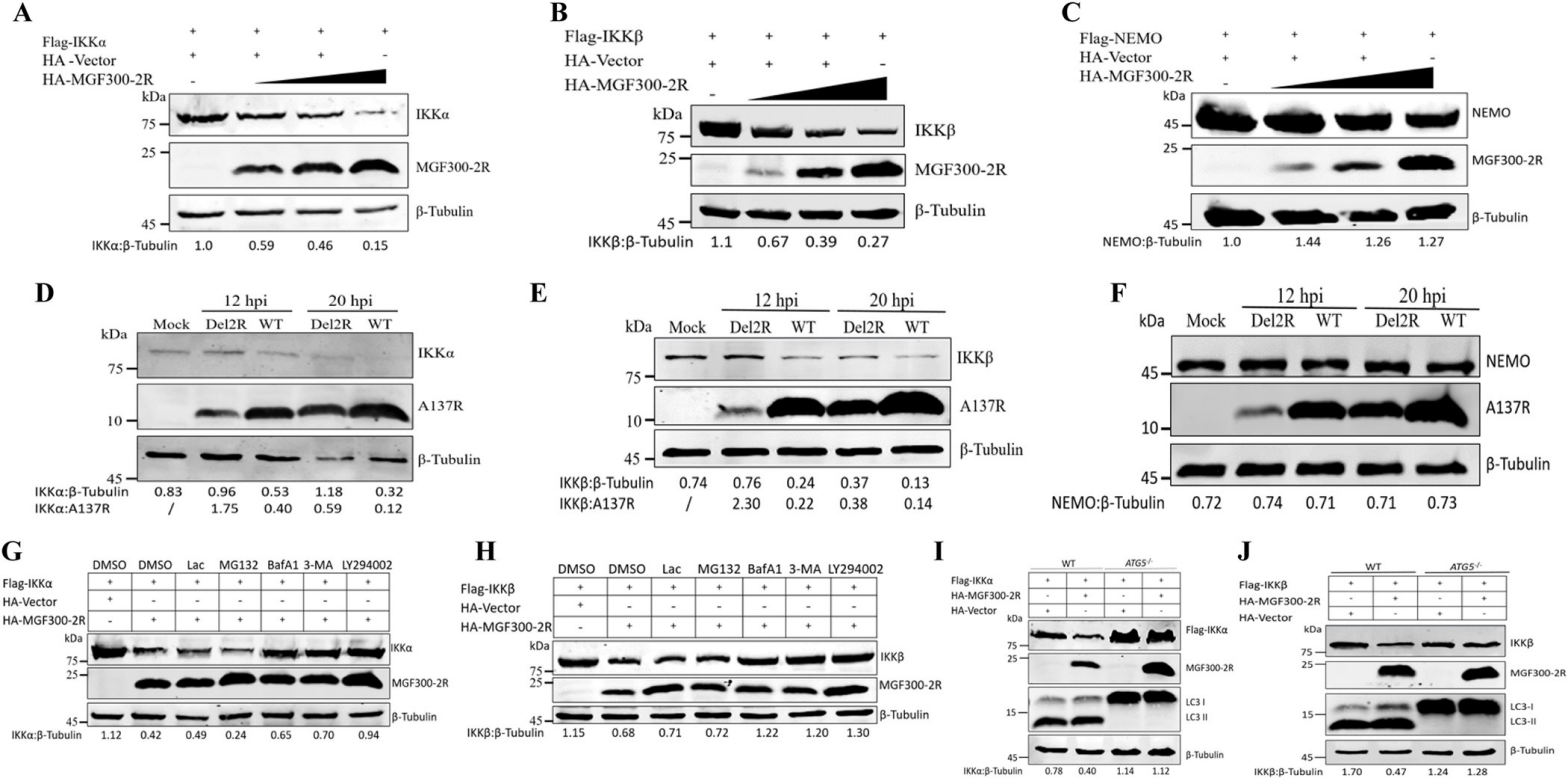
Autophagic degradation of IKK*α* and IKK*β* can be induced by the MGF300-2R protein. (A to C) HEK293T cells were cotransfected with each of pFlag-IKK*α*, pFlag-IKK*β*, or pFlag-NEMO, and an increasing amount of pHA-MGF300-2R (0, 1.0, 2.0, and 3.0 *μ*g) for 24 h. The cell lysates were analyzed by immunoblotting. (D to F) Analysis by western blotting of IKK*α* (D), IKK*β* (E), NEMO (F), and ASFV-A137R levels at 12 and 20 hpi in PAMs mock-infected or infected with ASFV-WT, or Del2R (MOI = 5). (G) HEK293T cells were cotransfected with pFlag-IKK*α* and pHA-MGF300-2R for 18 h, then the cells were treated with Lac (10 *μ*M), MG132 (10 mM), BafA1 (0.4 *μ*M), 3-MA (50 *μ*M), or LY294002 (20 mM) for 6 h. The cell lysates were analyzed by immunoblotting. (H) HEK293T cells were cotransfected with pFlag-IKK*β* and pHA-MGF300-2R for 18 h, then the cells were treated with Lac (10 *μ*M), MG132 (10 mM), BafA1 (0.4 *μ*M), 3-MA (50 *μ*M), or LY294002 (20 mM) for 6 h. The cell lysates were analyzed by immunoblotting. (I) Flag-IKK*α* and HA-MGF300-2R were cotransfected into the autophagy-related protein 5 (*ATG5*)-knockout (*ATG5^-/-^*) or wild-type (WT) HeLa cells for 36 h, and the expression of IKK*α* or MGF300-2R were analyzed by immunoblotting with the indicated antibodies. (J) Flag-IKK*β* and HA-MGF300-2R were cotransfected into the *ATG5^-/-^* or WT HeLa cells for 36 h, and the expression of IKK*β* or MGF300-2R were analyzed by immunoblotting with the indicated antibodies.

To investigate whether MGF300-2R affects the protein levels of IKK*α* and IKK*β*, we cotransfected HEK293T cells with pHA-MGF300-2R together with pFlag-NEMO, pFlag-IKK*α*, or pFlag-IKK*β*. Our results showed that the protein levels of IKK*α* (**Fig 4A**) and IKK*β* (**Fig 4B**) were markedly reduced in a dose-dependent manner, whereas the protein level of NEMO **(Fig 4C)** was not affected. Furthermore, we demonstrated that increased expression of MGF300-2R did not alter the transcriptional levels of IKK*α* (**S5A Fig**), IKK*β* (**S5B Fig**), and NEMO (**S5C Fig**). Interestingly, we observed that endogenous IKK*α* (**Fig 4D**) and IKK*β* (**Fig 4E**) were decreased in the PAMs infected with ASFV-WT at 12 and 20 hpi, whereas NEMO levels were not affected **(Fig 4F)**. However, compared with ASFV-WT, the degradation of endogenous IKK*α* and IKK*β* was diminished in the Del2R-infected PAMs at 12 and 20 hpi (**Fig 4D and 4E**). Additionally, the transcriptional levels of IKK*α* (**S5D Fig**), IKK*β* (**S5E Fig**), and NEMO (**S5F Fig**) were not affected in the PAMs infected with ASFV-WT or Del2R. These data show that absence of the endogenously expressed MGF300-2R during ASFV infection leads to increased IKK*α* and IKK*β* levels in PAMs. This confirms the role of MGF300-2R in inhibiting the NF-*κ*B-mediated innate immune response by inducing the degradation of IKK*α* and IKK*β*.

The ubiquitin-proteasome system and the autophagy-lysosome pathway are distinct mechanisms for intracellular protein degradation in eukaryotic cells [32]. Therefore, we further investigated which pathway is utilized by MGF300-2R to mediate the degradation of IKK*α* and IKK*β*. The HEK293T cells were transfected with MGF300-2R and IKK*α* or IKK*β* expression plasmids and treated with protease inhibitors Lac and MG132 or autophagy inhibitors, BafA1, 3-MA, or LY294002. The results showed that treatment with 3-MA, BafA1, and LY294002, but not MG132 and Lac, restored the degradation of IKK*α* (**Fig 4G**) and IKK*β* (**Fig 4H**). Moreover, the knockout of *ATG5*, an essential gene for autophagy activation [37,38], abrogated the MGF300-2R-mediated degradation of IKK*α* (**Fig 4I**) and IKK*β* (**Fig 4J**). Collectively, these results demonstrate that MGF300-2R promotes the degradation of IKK*α* and IKK*β* through the lysosomal degradation pathway.

### The MGF300-2R protein promotes the autophagic degradation of IKK*α* and IKK*β* by the cargo receptor TOLLIP

**Fig 5:**
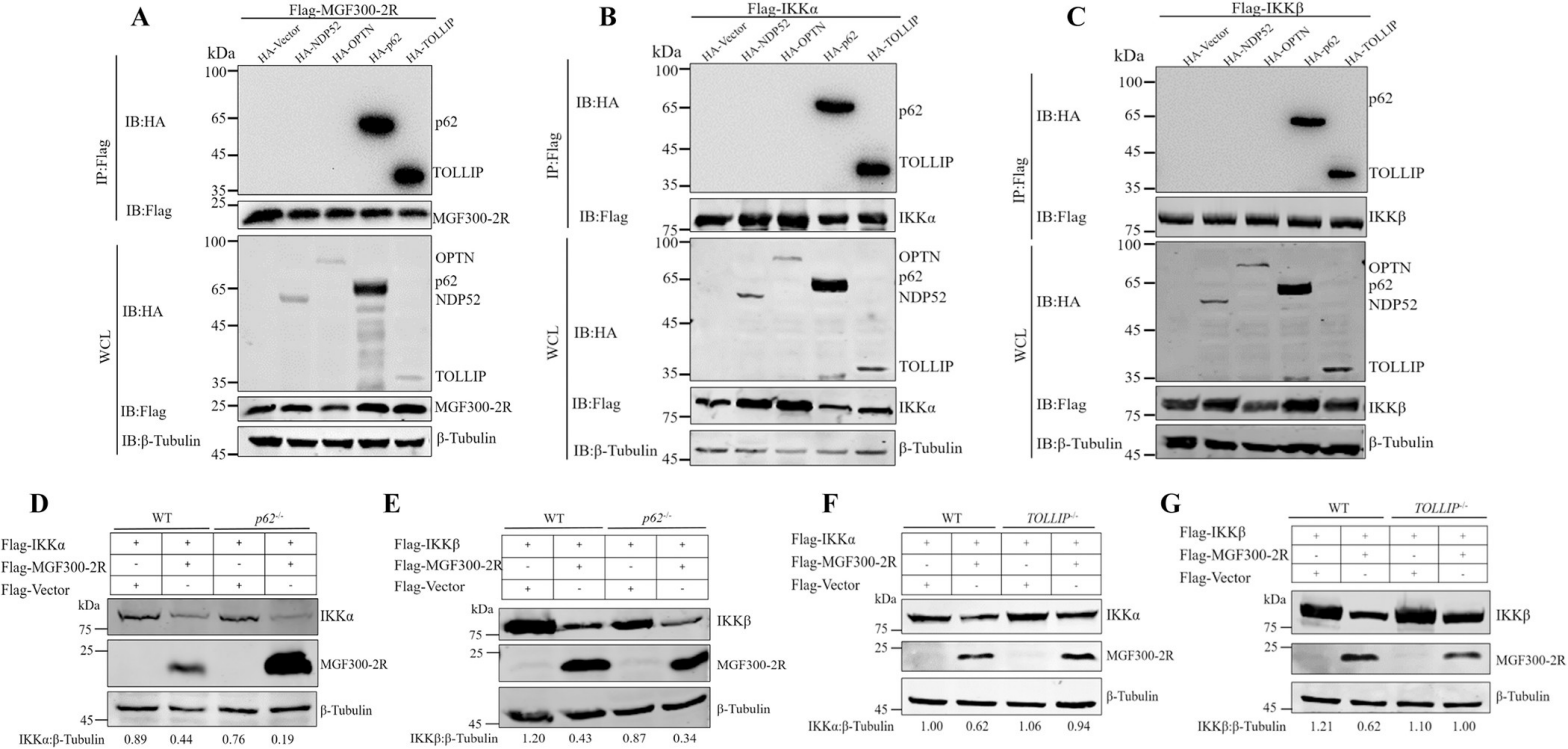
MGF300-2R promotes the autophagic degradation of IKK*α* and IKK*β* by the cargo receptor TOLLIP. (A to C) HEK293T cells were cotransfected with each of pFlag-MGF300-2R, pFlag-IKK*α*, pFlag-IKK*β*, and HA-tagged cargo receptors as indicated for 24 h, followed by immunoprecipitation with protein A/G beads. The WCL and IP precipitates were analyzed by immunoblotting with the indicated antibodies. (D and E) The *p62*-knockout (*p62^-/-^*) or wild-type (WT) HEK293T cells were cotransfected with Flag-IKK*α*, Flag-IKK*β*, and Flag-MGF300-2R for 36 h, and the expression of IKK*α*, IKK*β* or MGF300-2R was analyzed by immunoblotting. (F and G) The *TOLLIP*-knockout (*TOLLIP^-/-^*) or wild-type (WT) HEK293T cells were cotransfected with pFlag-IKK*α*, pFlag-IKK*β*, and pFlag-MGF300-2R for 36 h, and the expression of IKK*α*, IKK*β*, or MGF300-2R was analyzed by immunoblotting.

Previous experimental studies revealed that SARs play an essential role in defending against virus infection [30]. To investigate the mechanism of MGF300-2R-mediated autophagy-induced degradation of IKK*α* and IKK*β*, we hypothesized that MGF300-2R might act as a bridge between these proteins to a cargo receptor for autophagic degradation. To identify the specific cargo receptor responsible for MGF300-2R-induced degradation of IKK*α* and IKK*β*, we examined the interactions between MGF300-2R, cargo receptors (including p62, NDP52, TOLLIP, and OPTN), IKK*α* and IKK*β*. Co-IP assays revealed that MGF300-2R interacted with the cargo receptors p62 and TOLLIP (**Fig 5A**). Interestingly, we found that both p62 and TOLLIP interacted with IKK*α* (**Fig 5B**) or IKK*β* (**Fig 5C**). To investigate which receptor was required for the degradation of IKK*α* and IKK*β* mediated by MGF300-2R, we performed knockout experiments. Hence, HEK293T cells with *TOLLIP* knocked out (TOLLIP^−/−^ cells) were constructed using the CRISPR/Cas9 system **(S6A Fig)**. We found that the knockout of *p62* had a marginal effect on the degradation of IKK*α* **(Fig 5D)** and IKK*β* **(Fig 5E)**. In contrast, the degradation of IKK*α* **(Fig 5F)** and IKK*β* **(Fig 5G)** by MGF300-2R was significantly restored after the knockout of *TOLLIP* compared with the wild-type HEK293T cells. These data confirm that TOLLIP acts as a cargo receptor involved in the MGF300-2R-mediated autophagic degradation of IKK*α* and IKK*β*.

### The ASFV MGF300-2R protein promotes the K27-linked ubiquitination of IKK*α* and IKK*β*

**Fig 6:**
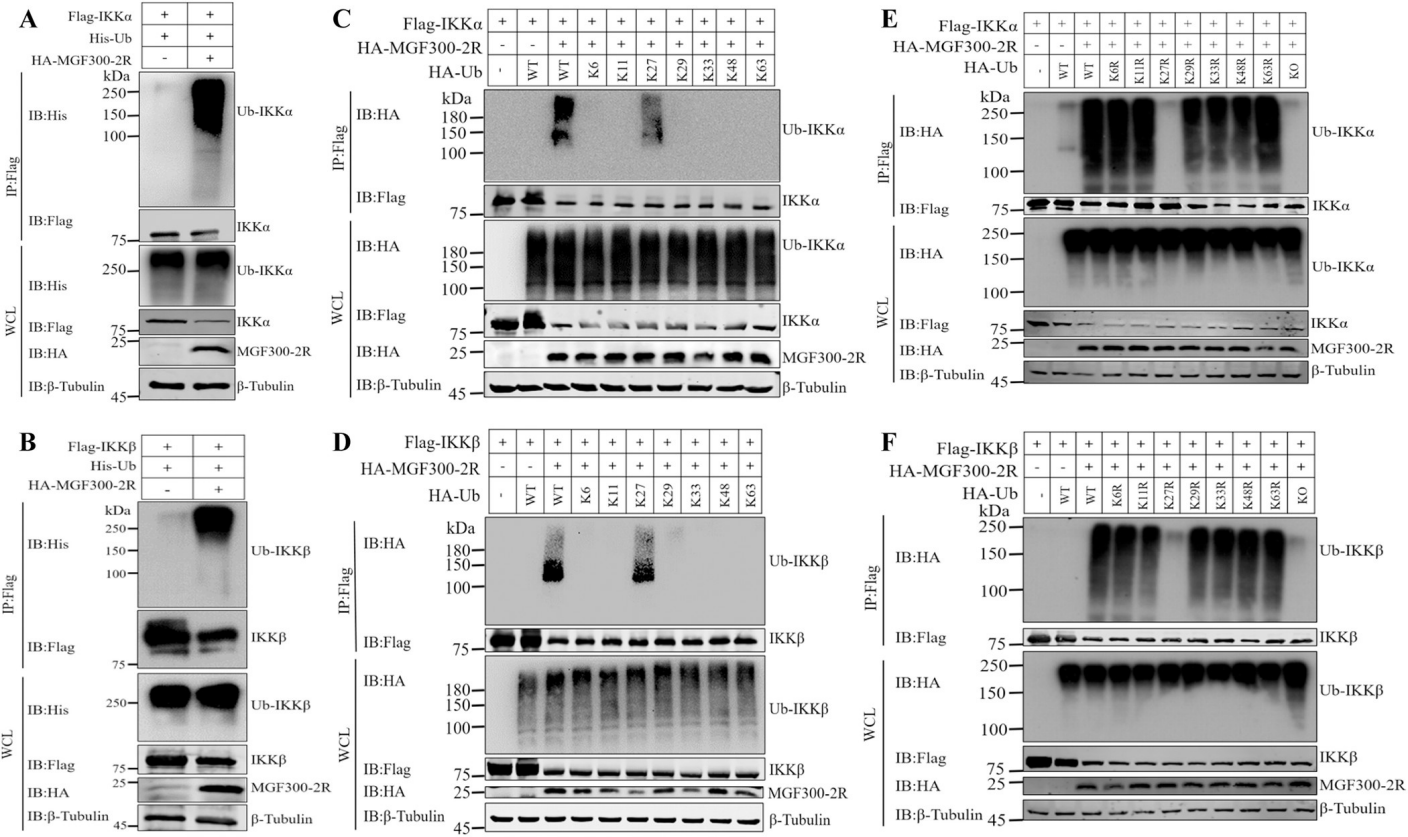
MGF300-2R facilitates the K27-linked ubiquitination and degradation of IKK*α* and IKK*β*. (A and B) MGF300-2R induces the ubiquitination of IKK*α* and IKK*β*. HEK293T cells were transfected with pFlag-IKK*α* or pFlag-IKK*β* and pHis-Ub together with pHA-MGF300-2R. At 24 hpt, cells were processed for IP with anti-Flag magnetic beads. WCLs and precipitated proteins were analyzed by western blotting with the indicated antibodies. (C and D) HEK293T cells were transfected with pFlag-IKK*α* or pFlag-IKK*β*, pHA-MGF300-2R, and pHA-Ub or its mutants (HA-K6, HA-K11, HA-K27, HA-K29, HA-K33, HA-K48, and HA-K63). At 24 hpt, cells were processed for IP with anti-Flag magnetic beads. WCLs and precipitated proteins were analyzed by western blotting with the indicated antibodies. (E and F) HEK293T cells were transfected with pFlag-IKK*α* or pFlag-IKK*β*, pHA-MGF300-2R, pHA-Ub or its mutants (HA-K6R, HA-K11R, HA-K27R, HA-K29R, HA-K33R, HA-K48R, and HA-K63R). At 24 hpt, cells were processed for IP with anti-Flag magnetic beads. WCLs and precipitated proteins were analyzed by western blotting with the indicated antibodies.

The selective autophagic pathway is involved the recognition of ubiquitinated substrates by SARs for delivery to autophagosomes for degradation via the selective autophagic pathway [29]. To examine whether MGF300-2R can promote the ubiquitination of IKK*α* and IKK*β*, we conducted a co-IP experiment. Cotransfection of pHis-Ub and pHA-MGF300-2R into HEK293T cells resulted in the induction of ubiquitination of IKK*α* and IKK*β* by MGF300-2R as shown in **Fig 6A and 6B**. To determine which type of polyubiquitination was responsible for the ubiquitination of IKK*α* and IKK*β*, we investigated the Ub chain linkage on each protein. By co-expression of IKK*α* or IKK*β*, MGF300-2R, and each of the seven Ub mutants, including K6, K11, K27, K29, K33, K48, and K63, that only harbor one of the seven lysine residues, we found that MGF300-2R promoted the polyubiquitination of IKK*α* (**Fig 6C**) and IKK*β* (**Fig 6D**) only in the presence of Ub and the mutant that retained K27, but not in the presence of mutants that retained K6, K11, K29, K33, K48, or K63. To confirm the role of K27 in IKK*α* and IKK*β* polyubiquitination, we generated seven Ub mutants, each with one of the seven lysine residues (K6, K11, K27, K29, K33, K48, and K63) mutated to arginine (R). We also generated a mutant (Ub-KO) in which all seven lysine residues were mutated to arginine. Co-IP analysis of MGF300-2R expression with different ubiquitin mutants showed that for both IKK*α* (**Fig 6E**) and IKK*β* (**Fig 6F**), K6R, K11R, K29R, K33R, K48R, and K63R lead to ubiquitination, while K27R did not show any significant difference compared with the Ub-KO. These results indicate that the K27-linked ubiquitination is responsible for the MGF300-2R-mediated autophagic degradation of IKK*α* and IKK*β*.

### MGF300-2R deletion attenuates ASFV virulence in pigs

**Fig 7:**
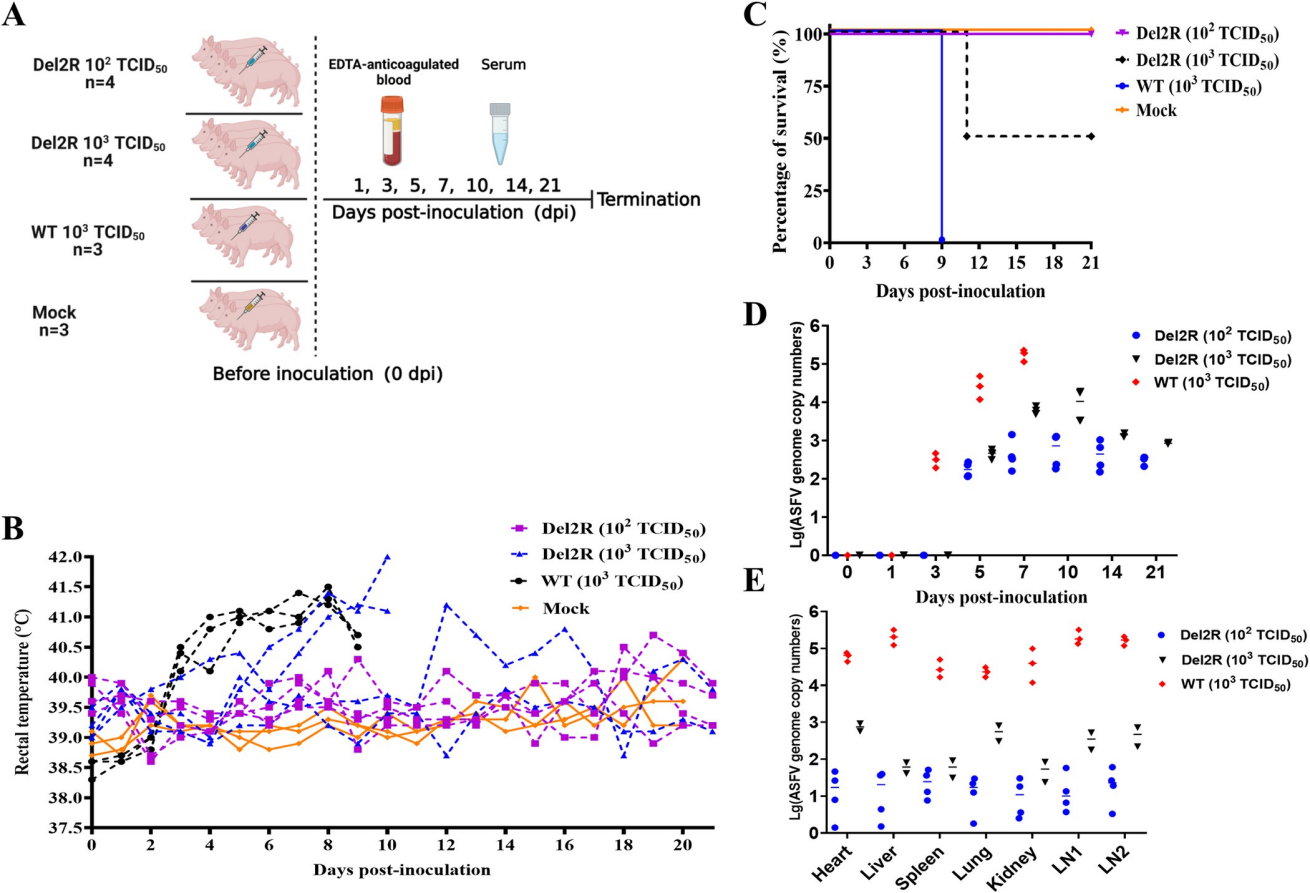
MGF300-2R is a virulence factor involved in the pathogenicity of ASFV. (A) The pigs were inoculated intramuscularly with Del2R (n = 4, 10^2.0^ or 10^3.0^ TCID_50_) or ASFV-WT (n = 3, 10^3.0^ TCID_50_) or mock inoculated intramuscularly with RPMI 1640 (n = 3, 1 mL), and the sera and blood were collected at 0, 1, 3, 5, 7, 14, and 21 days post-inoculation. The rectal temperature (B) and survival rates (C) of the different groups of pigs. (D) Viremia for the different groups of pigs infected with ASFV-WT or Del2R was detected by qPCR. (E) Viral loads in different tissues of the pigs inoculated with Del2R or ASFV-WT were detected by qPCR. LN1: submandibular lymph nodes, LN2: mesenteric lymph nodes.

To evaluate the viral pathogenicity of Del2R, we conducted challenge trials in pigs, a natural host of ASFV. The pigs were inoculated intramuscularly (i.m.) with Del2R at a dose of 10^2.0^ TCID_50_ or 10^3.0^ TCID_50_, ASFV-WT at a dose of 10^3.0^ TCID_50,_ or RPMI 1640 (Mock group) (**Fig 7A**). The disease progression was monitored for 21 d. Following the viral challenge, all three pigs inoculated with ASFV-WT developed fever at 3 dpi, which then gradually decreased to 41.5°C until died at 9 dpi (**Fig 7B**). In contrast, all the pigs in the 10^2.0^ TCID_50_ Del2R-inoculated and mock groups presented no obvious ASF-specific clinical signs or symptoms and had normal body temperatures during the 21-d observation period (**Fig 7B**). However, two pigs in the 10^3.0^ TCID_50_ Del2R-inoculated group developed a fever at 6 dpi and died at 11 dpi (**Fig 7B**), while the other two pigs had either a normal body temperature or a transient low fever before returning to normal. The pigs inoculated i.m. with 10^3.0^ TCID_50_ of ASFV-WT or Del2R showed a mortality of 100% (3/3) or 50% (2/4), respectively. By contrast, all the pigs infected with 10^2.0^ TCID_50_ of Del2R survived (**Fig 7C**). The viral genomic DNA in the pigs inoculated with ASFV-WT were detected as early as 3 dpi (10^2.5^ copies/mL), and gradually increased (10^5.2^ copies/mL) until all pigs died at 9 dpi. In contrast, all the pigs infected with Del2R had remarkably lower viral genomic DNA in the blood compared with those infected with ASFV-WT (**Fig 7D**). The tissue samples from the heart, liver, spleen, lung, kidney, submaxillary lymph node, and inguinal lymph node of the pigs infected with ASFV-WT showed high viral loads (10^4.3^ to 10^5.3^ copies/mL) (**Fig 7E**). In contrast, the results showed that tissue samples from the 10^2.0^ or 10^3.0^ TCID_50_ Del2R-inoculated pigs had lower (100 to 1000 folds) copies of viral DNA compared with the ASFV-WT-inoculated group (**Fig 7E**). Overall, these findings suggest that replication of Del2R in pigs is substantially attenuated compared with ASFV-WT, supporting our conclusion that MGF300-2R is a virulence factor and the virulence of Del2R is partially attenuated.

### Inflammatory response is induced by the MGF300-2R-deleted ASFV mutant in pigs

**Fig 8:**
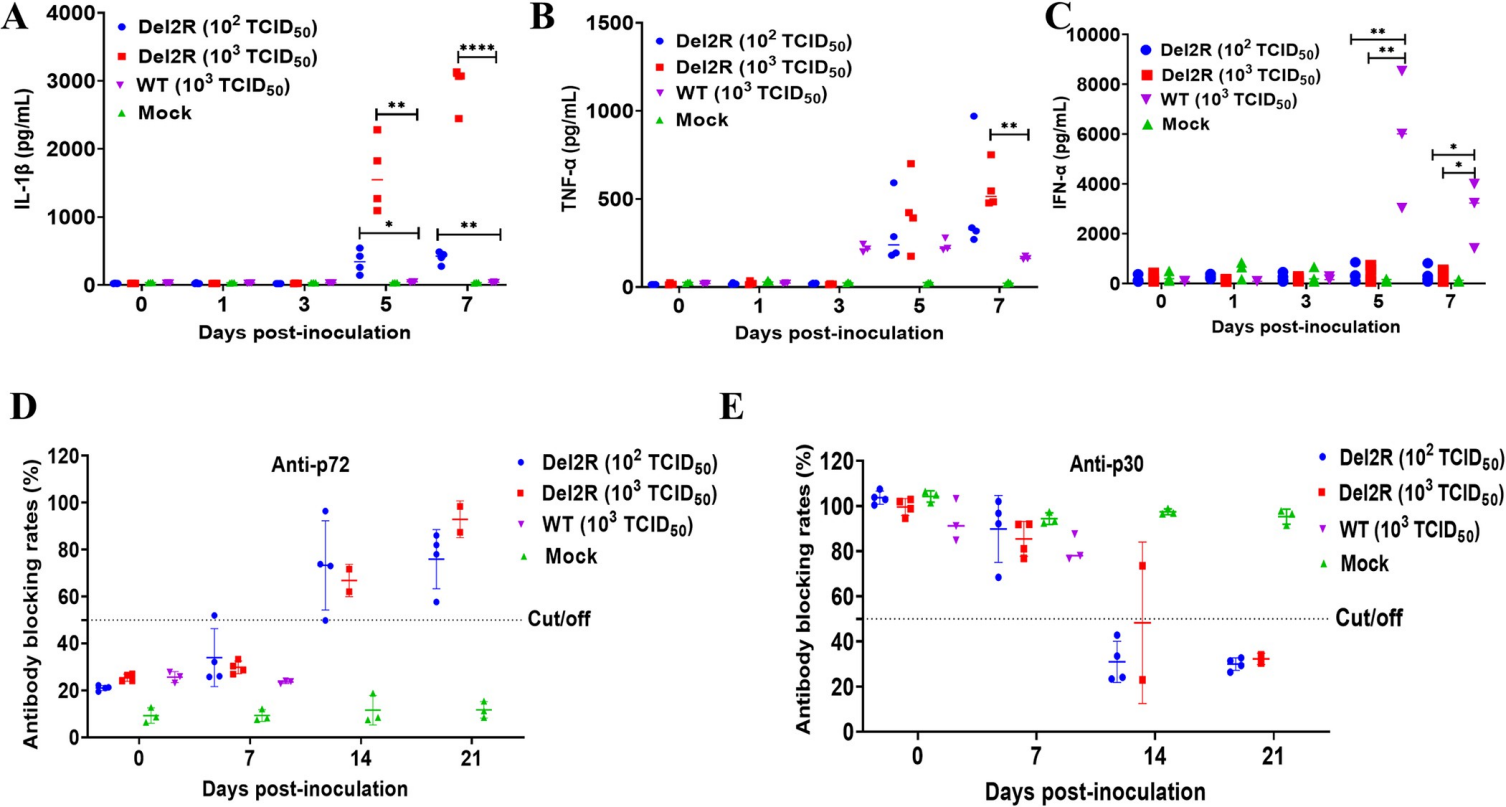
The *MGF300-2R*-deleted ASFV mutant (Del2R) induces higher pro-inflammatory cytokines and host antibody responses in pigs than does the wild-type ASFV (ASFV-WT). The protein levels of IL-1*β* (A), TNF-*α* (B), and IFN-*α* (C) in the serum samples were measured by commercial ELISA kits. Serum antibodies against p72 (D) or p30 (E) in the inoculated pigs were detected by ELISA. The results were shown as a blocking percentage. For the anti-p72 antibodies, the blocking rate above 50% was considered positive, while below 40% was considered negative; for the anti-p30 antibodies, the blocking rate below 40% was considered positive, while above 50% was considered negative. Samples with blocking between 40 and 50% were considered doubtful.

Our study has shown that infection with the attenuated recombinant Del2R induced high levels of IL-1*β* and TNF-*α* production in PAMs compared with infection with ASFV-WT (**Fig 2D and 2E**). Hence, we attempted to explore whether the Del2R infection enhances IL-1*β* and TNF-*α* production *in vivo*. We collected serum samples at 0, 1, 3, 5, and 7 dpi to evaluate the levels of IL-1*β*, IFN-*α*, and TNF-*α*. The pigs infected with Del2R produced significantly higher levels of IL-1*β* and TNF-*α* than did those infected with ASFV-WT at 5 and 7 dpi (**Fig 8A and 8B**). In contrast, the ASFV-WT inoculated pigs had significantly increased levels of IFN-*α* at 5 and 7 dpi (**Fig 8C**). These results demonstrated that the attenuated Del2R induced a higher inflammatory response in pigs compared with ASFV-WT.

Previous studies indicate that the levels of circulating antibodies, such as the antibodies against p30 and p72, are important parameters consistently associated with humoral immune responses and protection [4]. To gain an additional understanding of immune responses in the Del2R-infected pigs, we tried to detect ASFV-specific antibody response in the sera by ELISA. The p72 (a blocking rate above 50% is considered positive) and p30 (a blocking rate below 40% is considered positive) antibodies levels of the Del2R-infected pigs appear to increase from 7 dpi and reached peak titers at 21 dpi (**Fig 8D and 8E**), indicating that the partially attenuated Del2R induced better antibody responses. In contrast, no p72 and p30 antibodies were detected in the sera obtained from the mock and ASFV-WT inoculated pigs (**Fig 8D and 8E**). Taken together, these results suggest that MGF300-2R plays an important role in the evasion of antiviral innate immune responses and contributes to the virulence of ASFV.

## Discussion

Various combination of MGF360 (*9L*, *10L*, *11L*, *12L*, *13L*, and *14L*) and MGF505 (*1R*, *2R*, *3R*, and *7R*) genes deletion has been associated with reduced virulence and induction of different levels of protection in pigs against lethal homologous virulent ASFV challenge [10,39]. However, it remains unclear which critical *MGFs* genes have a greater effect on viral replication and pathogenicity than others. It has been shown that the cell-adapted strain ASFV-P121, with a 24.5-kb deletion in the LVR, lost the ability to replicate in PAMs [35]. Here, we further explored this deletion and found that it contains critical *MGFs* that affect ASFV replication in PAMs. By constructing a mutant virus (ASFV-DelLVR) with the same LVR deletion as ASFV-P121 based on the ASFV-WT, we were able to confirm this observation [35]. In this study, we identified a novel virulence factor, MGF300-2R, of ASFV and analyzed its contribution to viral pathogenesis in pigs. Significant differences were observed in the viral replication of ASFV-WT and the *MGF300-2R*-deleted ASFV mutant (Del2R) in pigs, indicating that attenuation of Del2R is correlated with reduced viral growth. Therefore, we conclude that MGF300-2R is a key factor for ASFV replication and virus pathogenesis.

To identify which *MGFs* located in the LVR have a greater effect on viral replication in PAMs, we generated six mutants with different combinations of *MGFs* deletions by homologous recombination. By comparing the replication kinetics of these mutant viruses with that of ASFV-WT in PAMs, we found that MGF300 family genes (*1L*, *2R*, and *4L*) significantly affect ASFV replication in PAMs **(Fig 1B)**. This study provided the first evidence of the role of the *MGF300-2R* gene, for which the function was unknown. We analyzed whether there are differences in binding, internalization, virus particle release, and viral factory formation between the Del2R and ASFV-WT, but no significant differences were observed **(S3 Fig)**. Recently, it has been found that the *MGFs* (*MGF110-9L*, *MGF360-9L*, and *MGF505-7R*) are involved in ASFV virulence and immune evasion [13–16]. We observed an increase in the expression levels of the NF-*κ*B mediated proinflammatory cytokines IL-1*β* and TNF-*α* in the Del2R-infected PAMs, as indicated using RNA-seq, RT-qPCR, and ELISA analysis **(Fig 2)**. Additionally, we detected a remarkable decrease in NF-*κ*B-regulated luciferase activity in cells expressing MGF300-2R. The signal transduction pathway(s) mediated by MGF300-2R to inhibitory NF-*κ*B was confirmed by inhibiting phosphorylation and nuclear translocation of p65. Therefore, we speculate that MGF300-2R may target the IKK complex and thereby inhibit the activation of the NF-*κ*B signaling pathway. Furthermore, we found that MGF300-2R interacts with IKK*α* and IKK*β* by co-IP, GST pulldown, and confocal microscopy. In addition, our findings suggest that MGF300-2R exerts its inhibitory effect on IL-1*β* and TNF-*α* production by selective autophagy to degrade IKK*α* and IKK*β*, which primarily occurs in the cytoplasm. The virus-encoded immunoregulatory proteins that are localized in the nucleus can interfere with the NF-*κ*B-mediated immune response either by outcompeting NF-*κ*B for KPNA binding and translocation into the nucleus or molecular mimicry [21]. Therefore, MGF300-2R may be a multifunctional protein that may employ a similar mechanism to inhibit NF-*κ*B activity. While further research is needed to explore this possibility, the functional data presented here provide an important clue for such investigations.

The presence of the NF-*κ*B antagonists A238L, F317L, and H240R further supports the importance of viral inhibition of NF-*κ*B activation in ASFV infection [24,25,27,40–45]. The ASFV-encoded protein A238L has been described to participate in regulating the inflammatory and immune response, mainly by controlling the NF-*κ*B signaling pathway [41]. The A238L protein is a homolog of the NF-*κ*B inhibitor I*κ*B, which can inhibit the production of TNF-*α* by inhibiting the transcriptional coactivator p300 [42–44]. Recent studies have shown that deletion of the *A238L* and *CD2v* genes attenuates the virulent ASFV strain Arm/07/CBM/c2 and protects pigs against virulent challenge [45]. The F317L protein inhibits the NF-*κ*B signaling by preventing activation of the IKK complex through interaction with IKK*β* and suppression of its phosphorylation [27]. Recently, the ASFV H240R protein has been shown to interact with NEMO and NLRP3, inhibiting subsequent the activation of the IKK complex and NLRP3 inflammasome [24,25,40]. In addition, the deletion of the *H240R* gene reduces the viral pathogenicity in pigs compared with its parental ASFV strain [40]. Similarly, MGF300-2R inhibits the NF-*κ*B signal transduction by preventing IKK complex activation via interaction with IKK*α* and IKK*β*.

IFNs and pro-inflammatory cytokines depending on NF-*κ*B regulation are essential for the antiviral immune response of the host [46]. As a counter-defense strategy, many viruses, such as VACV, ORFV, and HSV, have evolved multiple strategies to regulate the NF-*κ*B signaling, thereby creating a favorable environment for their replication. Some viral infections activate the NF-*κ*B pathway and promote viral replication. For example, HHV-6A-encoded U14 protein activates the NF-*κ*B signaling by interacting with the p65 thus promoting viral gene expression [47]. However, more often, viruses inhibit the NF-*κ*B-regulated inflammatory and immune responses at multiple levels by encoding various proteins during different stages of the viral life cycle. Large DNA viruses such as ASFV, Pseudorabies virus (PRV), VACV, ORFV, and HSV often encode multiple redundant proteins to regulate the NF-*κ*B signaling pathway and are involved in viral replication and pathogenicity [23, 41, 48]. A viral protein can play a regulatory role at different levels of the NF-*κ*B and IFN signaling pathways. For example, the virulence-associated protein MGF505-7R of ASFV can interact with IKK*α* and multiple adaptors of the IFN signaling pathway, including STING, IRF9, IRF3, JAK1, and JAK2 to inhibit IL-1*β* and IFN production, respectively [13–16,49]. Recently, numerous studies have demonstrated that the IFN inhibitors of ASFV are likely to influence viral replication and pathogenicity [50]. However, the actual role of the NF-*κ*B inhibitors of ASFV in viral pathogenicity remains poorly defined. Consistent with the results of *in vitro* studies, Del2R infection induced higher levels of IL-1*β* and TNF-*α* production with significantly reduced replication in pigs compared with ASFV-WT infection. The pigs infected with ASFV-WT all died at 9 dpi, while 50% of pigs challenged with Del2R survived (Fig 7C) at a dose of 10^3.0^ TCID_50_, suggesting that Del2R was dramatically attenuated. However, two pigs inoculated with 10^3.0^ TCID_50_ Del2R died at 11 dpi, which may have been due to excessive secretion of inflammatory cytokines. In addition, ASFV-WT inoculated pigs had significantly increased levels of IFN-*α* compared with the Del2R-infected pigs. This could be due to the higher level of replication of ASFV-WT than that of Del2R. Interestingly, the survival of the Del2R-infected pigs produced anti-p72 antibodies at 14 dpi **(Fig 8D)**, indicating the induction of a robust humoral immune response. These results indicate that the *MGF300-2R* gene may be a potential target for developing LAV candidates. Remarkably, single-gene deletion of the ASFV NF-*κ*B inhibitors affects viral pathogenesis and virulence variably. The diverse NF-*κ*B inhibitors encoded by ASFV, along with the possibility of overlapping or complementary functions, may explain this observation [13–16,40].

**Fig 9:**
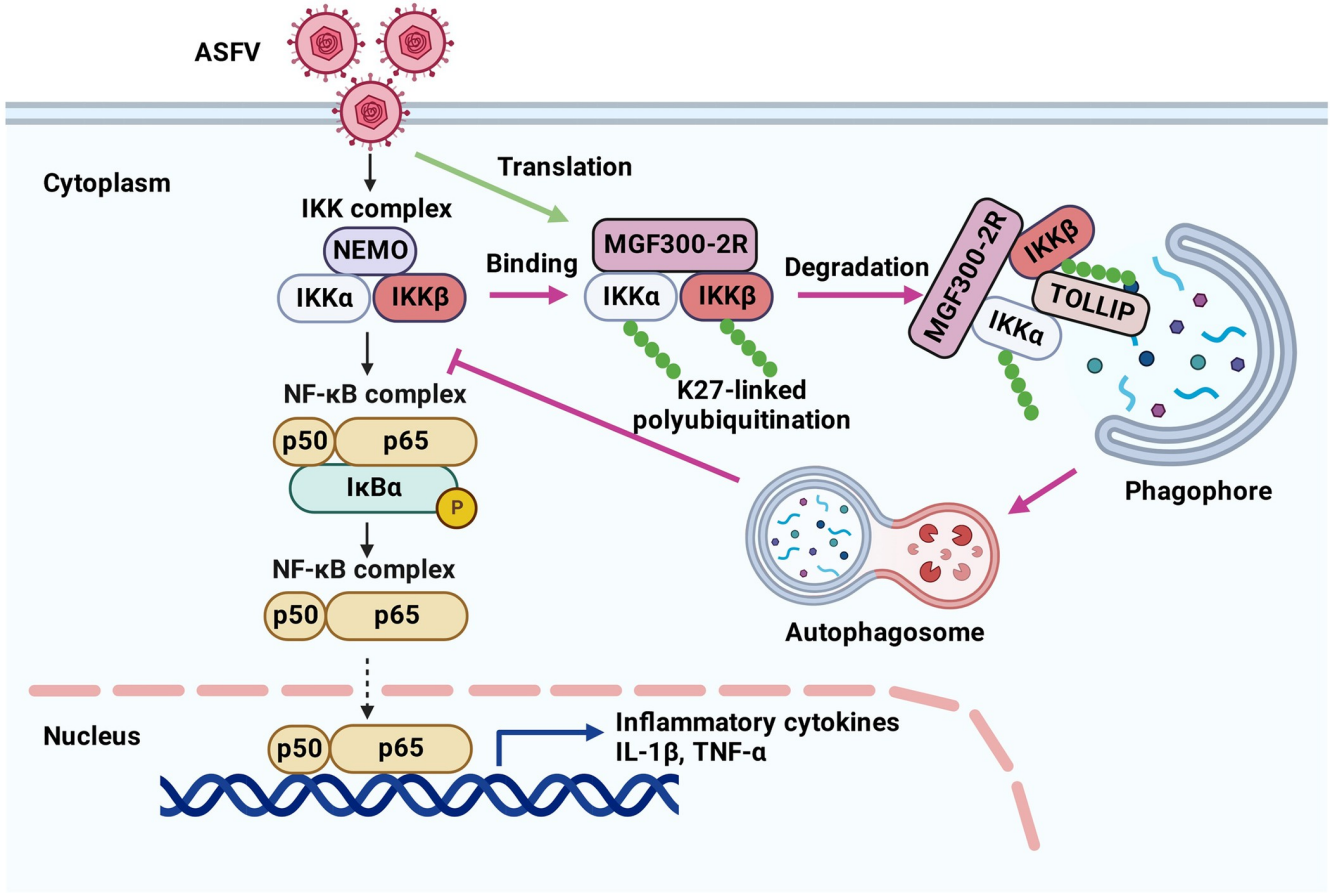
A working model for the regulation of the NF-*κ*B signaling pathway by the ASFV MGF300-2R protein. Black arrows indicate the NF-*κ*B signaling pathway. Upon infection, ASFV expresses the MGF300-2R protein, which interacts with both IKK*α* and IKK*β* and promotes the K27-linked ubiquitination of IKK*α* and IKK*β* for the TOLLIP-dependent autophagic degradation, serving as a suppressor to prevent the activation of the NF-*κ*B signaling pathway.

Selective autophagy is a powerful strategy in antiviral immunity of the host [30, 51,52]. Conversely, viruses have evolved various mechanisms to antagonize selective autophagy for successful infections. The L83L protein of ASFV promotes autophagic degradation of STING by recruiting TOLLIP, which inhibits type I IFN production and promotes viral replication [53]. Similarly, ASFV-encoded proteins I215L and A137R inhibit the production of type I IFN by targeting IRF9 and TBK1 for autophagic degradation, respectively [54,55]. Different from these reports, we discovered that MGF300-2R promotes the K27-linked ubiquitination of IKK*α* and IKK*β* and is degraded via the autophagic pathway, consequently inhibiting the production of inflammatory cytokines IL-1*β* and TNF-*α* **(Fig 9)**. TOLLIP and p62 have been reported to act as SAR and to be involved in degrading protein aggregates [30]. Here, we demonstrated that MGF300-2R protein induced the degradation of IKK*α* and IKK*β* through TOLLIP-mediated autophagy, which in turn negatively modulated the NF-*κ*B signaling and promoted ASFV replication. Further, to investigate whether MGF300-2R and TOLLIP are direct interactions, we performed GST pulldown experiments. The results showed that MGF300-2R and TOLLIP were indirect interactions **(S6B Fig)**. This suggests that MGF300-2R and IKK*α*, IKK*β*, and TOLLIP are likely to be degraded as a complex. This study is the first to report an association between the IKK complex and TOLLIP and provides evidence that TOLLIP plays a role in the innate immune response during ASFV infection.

Overall, in this study, we found that Del2R infection induces high levels of IL-1*β* and TNF-*α in vitro* and *in vivo*, and the MGF300-2R plays a critical role in inhibiting the production of inflammatory cytokines. Pathogenesis studies have been conducted on ASFV in pigs using a modified strain called Del2R, which lacks a single NF-*κ*B inhibitor gene known as *MGF300-2R*. These studies have shown a significant reduction in pathogenicity and a marked increase in the production of inflammatory cytokines. Mechanistically, the MGF300-2R protein interacted with IKK*α* and IKK*β* and mediated the degradation of IKK*α* and IKK*β* through the cargo receptor TOLLIP-mediated selective autophagy pathway **(Fig 9)**. Our findings help us understand the functions of MGF300-2R and its role in viral pathogenesis, which might help the rational design of LAVs to control ASF.

## Materials and methods

### Ethics statements

All the experiments involving the ASFV HLJ/18 and Del2R in this study were performed in the enhanced biosafety level 3 facilities (BSL3) in the Harbin Veterinary Research Institute (HVRI) of the Chinese Academy of Agricultural Sciences (CAAS) approved by the Ministry of Agriculture and Rural Affairs, China. This study was conducted in compliance with the Animal Welfare Act and Guide for the Care and Use of Laboratory Animals, approved by the Laboratory Animal Welfare Committee of HVRI.

### Cells and viruses

Human embryonic kidney 293T (HEK293T) and human cervical carcinoma cell line HeLa were cultured in Dulbecco’s modified Eagle’s medium (DMEM, Sigma-Aldrich) supplemented with 10% fetal bovine serum (FBS) at 37°C in a 5% CO_2_ incubator. *ATG5*-knockout (KO) HeLa cells and *p62*-KO HEK293T cells were reported [32]. *TOLLIP*-KO HEK293T cells were generated using the CRISPR/Cas9 system with sgRNAs (*sgTOLLIP*: 5’-CAGCCGTGTTCAGGCGACTC-3’) inserted into the plasmid pX458 vector. PAMs were maintained in RPMI 1640 medium with L-glutamine (Thermo Fisher Scientific, USA) supplemented with antibiotics (100 U/mL penicillin and 100 mg/mL streptomycin) and 10% FBS at 37°C a 5% CO_2_ incubator. The ASFV HLJ/18 strain (ASFV-WT) (GenBank accession number MK333180.1) was isolated as described previously [56].

### Antibodies and reagents

Rabbit anti-*β*-tubulin (A12289), mouse anti-HA (AE008), anti-Flag (AE005), anti-His (AE003), anti-GST (AE001), and rabbit anti-IKK*γ* (A0917) antibodies were purchased from Abclonal. Rabbit anti-IKK*α* (61294S), anti-IKK*β* (2678S), rabbit anti-phospho-NF-*κ*B p65 (Ser536) (3033S), rabbit anti-phospho-I*κ*B*α* (Ser32) (2859S), rabbit anti-NF-*κ*B p65 (6956S) and mouse anti-*κ*B*α* (4814S) antibodies were purchased from Cell Signaling Technology. Rabbit anti-TOLLIP (11315-1-AP), anti-LC3 (14600-1-AP), anti-lamin B1 (12987-1-AP), and mouse anti-GAPDH (60004-1-Ig) antibodies were purchased from Proteintech. HRP-conjugated goat anti-mouse IgG (115-035-003) and anti-rabbit IgG (111-035-003) antibodies were purchased from Jackson ImmunoResearch. Alexa Fluor 488-conjugated goat anti-mouse IgG (H+L) (A11029) and anti-rabbit IgG (H+L) (A11008), Alexa Fluor 647-conjugated donkey anti-mouse IgG (H+L) (A31571) and anti-rabbit IgG (H+L) (A31573), LICOR IRDye 800CW goat anti-rabbit IgG (H+L) (926–32211) and goat anti-mouse IgG (H+L) (926–32212) antibodies were purchased from Thermo Fisher Scientific. Anti-FLAG(R) M2 magnetic beads (M8823) and anti-HA agarose (A2095) were purchased from Sigma-Aldrich. Anti-A137R polyclonal antibody was prepared by immunizing the rabbits with ASFV protein A137R [48]. 4’, 6-Diamidino-2-phenylindole (DAPI) (C006) were purchased from Solarbio. Bafilomycin A1 (BafA1) (sc-201550A) was purchased from Santa Cruz Biotechnology. Carbobenzoxy-leu-leu-leucinal (MG132) (474790), lactacystin (L6785), and 3-methyladenine (3-MA) (M9281) were purchased from Sigma-Aldrich. LY294002 (PHZ1144) was purchased from Thermo Scientific. TNF-*α* recombinant protein (300-01A-50) was purchased from PeproTech.

### Construction of plasmids

The ASFV *MGF300-2R* gene was optimized for the expression in human cells by AZENTA (Suzhou, China), and then cloned into the vector pCMV-HA or pCMV-Flag vectors, resulting in pHA-MGF300-2R and pFlag-MGF300-2R, respectively. The *MGF300-2R* gene was inserted into the pGEX-6P-1 vector, generating pGST-MGF300-2R by AZENTA (Suzhou, China). The plasmids, including pFlag-cGAS, pFlag-STING, pIFN-*β*-Fluc, pISRE-Fluc, pNF-*κ*B-Fluc, and pTK-Rluc, plasmids were described previously [55]. The pFlag-p62 (RC203214), pFlag-TOLLIP (RC200227), pFlag-OPTN (RC202470), and pFlag-NDP52 (RC203843) were purchased from Origene and then mutated using PCR site-directed mutagenesis to generate pHA-p62, pHA-TOLLIP, pHA-OPTN, and pHA-NDP52, respectively. The porcine *IKKα*, *IKKβ*, and *NEMO* genes were cloned into the pCMV-Flag to generate pFlag-IKK*α*, pFlag-IKK*β*, and pFlag-NEMO, respectively. The nucleotide sequences of all inserts in the plasmids were verified by the Sanger DNA sequencing. The primers for amplification of the plasmids were listed in **S1 Table.**

### Generation of the *MGFs*-deleted ASFV mutant

Nine recombinant *MGFs*-deleted ASFV mutants (**Fig 1A**) were generated by homologous recombination between transfer vectors containing left and right flanking regions of the gene(s) to be deleted and *EGFP* reporter genes under the control of the ASFV *p72* promoter and the parental virus. The transfer vectors were constructed as described previously [35]. To confirm that the *MGFs* had been deleted accurately, the genomes of the *MGFs*-deleted ASFV mutants (DelLVR, Del1L, Del2R, and Del4L) were extracted from the infected PAMs using QIAamp blood mini kit (QIAGEN, 51104). The Illumina libraries and sequencing were performed by Novogene (Beijing, China) using the NEBNext ultra DNA library prep kit (USA) following the manufacturer’s recommendations. We first trimmed the adapter sequences and removed low-quality reads from the raw data using Trimmomatic v0.36 [57]. Then, the clean reads were mapped with the reference ASFV genome sequence (MK333180.1) using Bowtie2 v2.3.5.1 with default parameters [58]. Subsequently, alignment files were used for the variant calling process with Samtools 1.3.1 and Varscan 2.4.3 [59,60]. Single nucleotide polymorphisms (SNPs), insertion and deletion (INDEL) were analyzed and compared with the reference ASFV genome sequence. The functional effects of the SNPs and INDELs were identified using SnpEff v4.0 [61]. All viruses were analyzed by PCR to identify the recombinants by distinguishing wild-type and *MGFs*-deleted ASFVs. To verify that all the final recombinant viruses harbored the correct sequences, the PCR fragments spanning the deleted *MGFs* region were sequenced. The primers for the construction of the transfer vectors and PCR were listed in **S1 Table**.

### One-step viral growth kinetics

Comparative growth curve assays between the *MGFs*-deleted ASFV mutants and ASFV-WT were performed in PAMs. The PAMs were cultured in 24-well cell culture plates and infected with the above ASFV mutants at a multiplicity of infection (MOI) of 5 and harvested at 2, 12, 24, 72, 120, and 168 hours post-infection (hpi). Virus titers at each time point were determined as previously described [34].

### Transmission electron microscopy (TEM), and virus binding and entry assays

PAMs were seeded in 12-well cell culture plates and allowed to grow for 12 h before being then infected with either ASFV-WT or the *MGFs*-deleted ASFV mutants at a MOI of 5. At 24 hpi, the cells were fixed with 2% glutaraldehyde and prepared for TEM observation following established protocols [25]. The ASFV binding and entry assays were conducted in PAMs described previously [25].

### RNA-seq analysis and quantitative real-time PCR (qPCR)

For the RNA-seq, PAMs were cultured in 12-well cell culture plates, infected with the *MGF300-2R*-deleted ASFV mutant (Del2R) or ASFV-WT in triplicate at a MOI of 5 for 12 or 20 h, and collected in TRIzol reagent (Invitrogen, 15596026CN). RNA-seq analysis was performed as described previously [55]. Total RNAs from the PAMs infected with Del2R or ASFV-WT was isolated using a cell total RNA isolation kit (BioFlux, BSC52M1), and cDNA was synthesized using FastKing gDNA Dispelling RT SuperMix (Tiangen, KR118-02). The resulting cDNAs were used as templates for RT-qPCR using SYBR Premix Ex Taq (TaKaRa, RR390B) to assess the expression levels of *IL-1β*, *IFN-β*, *TNF-α*, *IKKα*, *IKKβ*, and *NEMO* genes. Glyceraldehyde-3-phosphate dehydrogenase (GAPDH) was used as a reference gene for normalization. The qPCR primers used in this study are listed in **S1 Table**. The relative abundance of each gene was calculated using the comparative cycle threshold (2^-ΔΔCT^) method.

### Detection of inflammatory cytokines

PAMs were seeded in 24-well cell culture plates for 12 h, then infected with ASFV-WT or Del2R for 1 h and rinsed twice with PBS. The infected cells were cultured in the fresh RPMI 1640 medium for 12 and 20 h. The concentrations of IL-1*β* (Ray Biotech, ELP-IL1b-1), TNF-*α* (Ray Biotech, ELP-TNFa-1), and IFN-*α* (Ray Biotech, ELP-IFNa-1) in the cell supernatants were measured by enzyme-linked immunosorbent assay (ELISA) kits according to the manufacturer’s instructions. The cells were collected for RT-qPCR analysis as described above, and the mock-infected cells were included as a negative control.

### Transfection

HEK293T and HeLa cells were transfected using linear 25-kDa polyethyleneimine (PEI) (Polysciences, 23966–2). Briefly, a transfection mixture consisting of 3 *μ*g of the indicated plasmid and 9 *μ*L of PEI (stock solution at 1 mg/mL) in 300 *μ*L of Opti-MEM (Invitrogen, 31985070) was used to transfect cells of 80% confluence in 6-well cell culture plates. After a 20-min incubation at room temperature, the transfection mixture was added to the cells and incubated for 24–36 h. PAMs were transfected using X-tremeGENE HP (Roche, 6366546001) according to the manufacturer’s instructions.

### Laser confocal microscopy

HEK293T cells were seeded on a poly-L-lysine-coated glass-bottom cell culture dish (NEST, 801001) and transfected with the indicated expressing plasmids using PEI. At 24 hours post-transfection (hpt), the cells were fixed with 4% paraformaldehyde for 20 min at room temperature, followed by permeabilized with 0.1% triton X-100 (Sigma-Aldrich, V900502) in PBS for 20 min, and then blocked with 5% bovine serum albumin (BSA). Specific primary antibodies were added and incubated for 2 h. After washing with PBS for five times, the cells were stained with the Alexa Fluor-conjugated goat anti-mouse IgG or goat anti-rabbit IgG secondary antibodies for 1 h at room temperature. The cells were washed with PBS for three times and then incubated with DAPI for 5 min for nuclear staining. After three washing steps with PBS, the cells were observed under a confocal laser scanning microscopy with Airyscan (Zeiss, LSM800), and the most representative images were selected for presentation. Colocalization analysis between MGF300-2R and IKK*α* or IKK*β* was performed by calculating the Pearson’s correlation coefficients using the coloc2 tool of ImageJ/FIJI.

### Western blotting, co-immunoprecipitation (co-IP) and GST pull-down assays

HEK293T cells were harvested and lysed in ice-cold RIPA lysis buffer (Sigma-Aldrich, R0278) supplemented with a protease inhibitor cocktail (Roche, 4693116001). The cell lysates were boiled in SDS-polyacrylamide gel electrophoresis (SDS-PAGE) loading buffer (Solarbio, P1040) and resolved by SDS-PAGE. Separated proteins were transferred onto polyvinylidene fluoride membranes, followed by blocking with 5% skim milk in Tris-buffered saline with 0.05% tween 20 (TBST) for 1 h at room temperature. The membranes were then probed by appropriate primary and secondary antibodies. The signals were detected using an Odyssey imaging system.

The transfected HEK293T cells were lysed in RIPA lysis buffer for 30 min on ice. After removal of nuclei through low-speed centrifugation and collecting 100 *μ*L of the sample as input, the remaining 800-μL lysates were incubated with the appropriate antibody, followed by the addition of anti-Flag M2 magnetic beads or anti-HA agarose and rotated at 4°C overnight. After incubation, the beads were collected using a magnetic separator and washed three times with PBS. The bound proteins were subjected to western blotting using appropriate antibodies. Densitometric analysis of the blots was performed with ImageJ/FIJI as previously described [32]. Levels of target proteins such as MGF300-2R were normalized to the loading control *β*-tubulin.

Glutathione S-transferase (GST) pulldown assay was performed as described previously [55]. Briefly, the purified recombinant GST-MGF300-2R or GST protein was incubated with the lysates from the HEK293T cells transfected with pHA-TOLLIP, pFlag-IKK*α* or pFlag-IKK*β* at 4°C for 12 h. The proteins pulled down by glutathione-sepharose 4B resin (GE Healthcare, 17-0756-01) were analyzed by western blotting.

### Dual-luciferase reporter assay

HEK293T cells were cotransfected with the reporter plasmids (pIFN-*β*-Fluc, pISRE-Fluc, pIRF3, and pNF-*κ*B-Fluc) and an internal control vector pTK-Rluc along with or without indicated expressing plasmids using X-tremeGENE HP transfection reagent. At 24 hpt, the cells were washed with PBS for three times. The passive lysis buffer was added for 20 min at 4°C with gentle shaking. The luciferase activities were then determined by a dual-luciferase reporter assay system (DLR) (Promega, E1910), according to the manufacturer’s instructions. To analyze the effects of the inhibitors, the transfected cells were left untreated or treated with 10 ng of TNF-*α* as an inducer of NF-*κ*B. The luciferase activity (Fluc/Rluc) was calculated by dividing the firefly luciferase activity by the *Renilla* luciferase activity.

### NF-*κ*B-p65 nuclear translocation assay

HEK293T cells were transfected with expressing plasmid pFlag-MGF300-2R for 24 h, followed by being washed twice with PBS and cultured in indicated medium. The cells were stimulated with TNF-*α* (20 ng/mL) for 1 h. The separation of cellular fractions was performed by a Minute plasma membrane protein isolation and cell fractionation kit (Invent Biotechnologies, SM-005). The resulting supernatants were collected as a nuclear extract. Tubulin and lamin B1 were used as cytoplasm and nucleus markers, respectively. The nuclear and cytoplasmic compartments were examined by western blotting. To investigate the effect of MGF300-2R on nuclear translocation of the NF-*κ*B-p65 following ASFV infection. PAMs were mock-infected or infected with ASFV-WT or Del2R (MOI = 5). At 24 hpi, the separation of cellular fractions of ASFV-infected PAMs was performed as described above.

### Pig inoculation experiments

Fourteen healthy specific-pathogen-free piglets, aged 4 weeks and weighing between 11.5 and 13.5 kg, were obtained from the Laboratory Animal Center of the HVRI. Back titrations were performed to confirm the titers of the Del2R or ASFV-WT inoculum. The piglets were randomly divided into four groups and inoculated i.m. with ASFV to investigate the pathogenesis of the Del2R, with four piglets inoculated with Del2R (10^2.0^ TCID_50_/piglet); 4 piglets inoculated with Del2R (10^3.0^ TCID_50_/piglet); three piglets inoculated with ASFV-WT (10^3.0^ TCID_50_/piglet); three piglets inoculated with RPMI 1640 (1 mL/piglet). Daily monitoring was conducted for each pig, recording rectal temperatures and the clinical signs for each pig, including lethargy, anorexia, depression, vomiting, fever, skin hemorrhages, bloody diarrhea, and joint swelling. EDTA-anticoagulated blood and sera were collected from all pigs at 0 1, 3, 5, 7, 10, 14, and 21 days post-infection (dpi) for inflammatory cytokines detection by ELISA kits as described above and virus load detection by qPCR, respectively. Anti-p72 (INGENASA, 11.PPA.K.3/5) and -p30 (ID.VET, ASFC-5P) antibodies were measured using commercial ELISA kits following the manufacturer’s instructions. At 21 dpi, all surviving piglets were euthanized and seven different tissues and organs, including the heart, liver, spleen, lung, kidney, submandibular lymph nodes, and mesenteric lymph nodes were obtained from each necropsied pig, for viral load detection by qPCR as described previously [34].

### Statistical analysis

An unpaired two-tailed *t* test was used to determine statistical significance and performed in GraphPad Prism version 8.0.0 (GraphPad Software, San Diego, California, USA). Data are presented as the mean ± SD of three independent experiments. *P* ≥ 0.05 was considered statistically non-significant (ns); *P* < 0.05 was considered statistically significant (* *P* < 0.05; ** *P* < 0.01; *** *P* < 0.001).

## Supporting information

S1 Fig Generation of ASFVs with deletion of *MGFs*. (A) The PCR results of amplifying the genomic segment containing the targeted genes. NC: negative control. Complete genome coverage plot using the NGS reads of the recombinant viruses Del1L (B), Del2R (C), and Del4L (D) mapped against the ASFV HLJ/18 genome. The read coverage values of Del1L, Del2R, and Del 4L were calculated using Samtools and plotted using the Rstudio software (v3.6.1).
(TIF)

S2 Fig Identification of the nuclear localization signal in the MGF300-2R protein. (A) Schematic representation of truncation mutants of MGF300-2R. Dashed lines indicate the range of deleted amino acid relative to the MGF300-2R protein. (B) HEK293T cells were transfected with the mutant-expressing plasmids for 24 h, and the expression of the indicated mutants was detected by western blotting. (C) HEK293T cells were transfected with the mutant-expressing plasmids for 24 h and the subcellular localization of the indicated mutants was examined by confocal microscopy. Bars, 20 *μ*m. (D) Twenty cells were counted in randomly selected view fields two times, and the MGF300-2R nuclear localization ratio was evaluated.
(TIF)

S3 Fig The MGF300-R protein is not required for ASFV binding, internalization, the release of virions, or viral factory morphogenesis. PAMs were infected with Del2R or ASFV-WT (MOI = 5). Binding, internalization (A), and release of virions (B) were analyzed. (C) Morphology of virus factories (red circles) in the Del2R or ASFV-WT infected PAMs (MOI = 5) at 24 hpi. Bars, 1 *μ*m. (D) PAMs were infected with Del2R or ASFV-WT (MOI = 5) and fixed with 2% glutaraldehyde at 24 hpi. The viral budding process (arrowheads) was analyzed by transmission electron microscopy. Bars, 2 *μ*m.
(TIF)

S4 Fig MGF300-2R does not affect the IFN signaling pathway. (A and B) PAMs were either mock infected or infected with Del2R or ASFV-WT (MOI = 5). At 12 and 20 hpi, the mRNA levels of IFN-*α* (A) in the cell lysates were determined by RT-qPCR and the production of IFN-*α* (B) in the cell culture supernatants was detected by ELISA kits. (C to E) HEK293T cells were cotransfected with increasing amounts of the expressing plasmid pHA-MGF300-2R (0.5, 1, and 1.5 *μ*g) along with IFN-*β* (C), IRF3 (D), or ISRE (E) promoter reporter and the luciferase activities were measured. (F) The protein expression of MGF300-2R, cGAS, and STING was analyzed by western blotting.
(TIF)

S5 Fig MGF300-2R does not affect the transcription of IKK*α*, IKK*β*, and NEMO. HEK293T cells were transfected with increasing amounts of the expressing plasmid pHA-MGF300-2R (0.5, 1, and 1.5 *μ*g), and the mRNA levels of IKK*α* (A), IKK*β* (B), and NEMO (C) were determined by RT-qPCR. PAMs were either mock infected or infected with Del2R or ASFV-WT (MOI = 5). At 12 and 20 hpi, the mRNA levels of IKK*α* (D), IKK*β* (E), and NEMO (F) in the cell lysates were determined by RT-qPCR.
(TIF)

S6 Fig Identification of the TOLLIP-knockout HEK293T cells. (A) TOLLIP expression was determined by western blotting with antibodies against TOLLIP and *β*-tubulin. (B) HEK293T cells were transfected with the expressing plasmid pHA-TOLLIP or pFlag-IKK*β* for 36 h and lysed with NP-40 buffer. The purified GST or GST-MGF300-2R protein was used to pull down the TOLLIP or IKK*β* in the lysates and analyzed by western blotting with the indicated antibodies.
(TIF)

S1 Table The primers used in this study. (XLSX)

S2 Table Illumina sequencing statistics and variation analysis of the MGF300-1L- (Del1L), MGF300-2R- (Del2R), and MGF300-4L-deleted (Del4L) ASFV mutants. (XLSX)

S3 Table The differentially expressed genes between the ASFV-WT- and Del2R-infected PAMs at 12 and 20 hpi. (XLSX)
