# Supplementary figures and images for: The MGF300-2R protein of African swine fever virus is associated with viral pathogenicity by promoting the autophagic degradation of IKKα and IKKβ through the recruitment of TOLLIP
Source: PLoS Pathog. 2023 Aug 11;19(8):e1011580. doi: 10.1371/journal.ppat.1011580 (PMC10446188; doi:10.1371/journal.ppat.1011580)

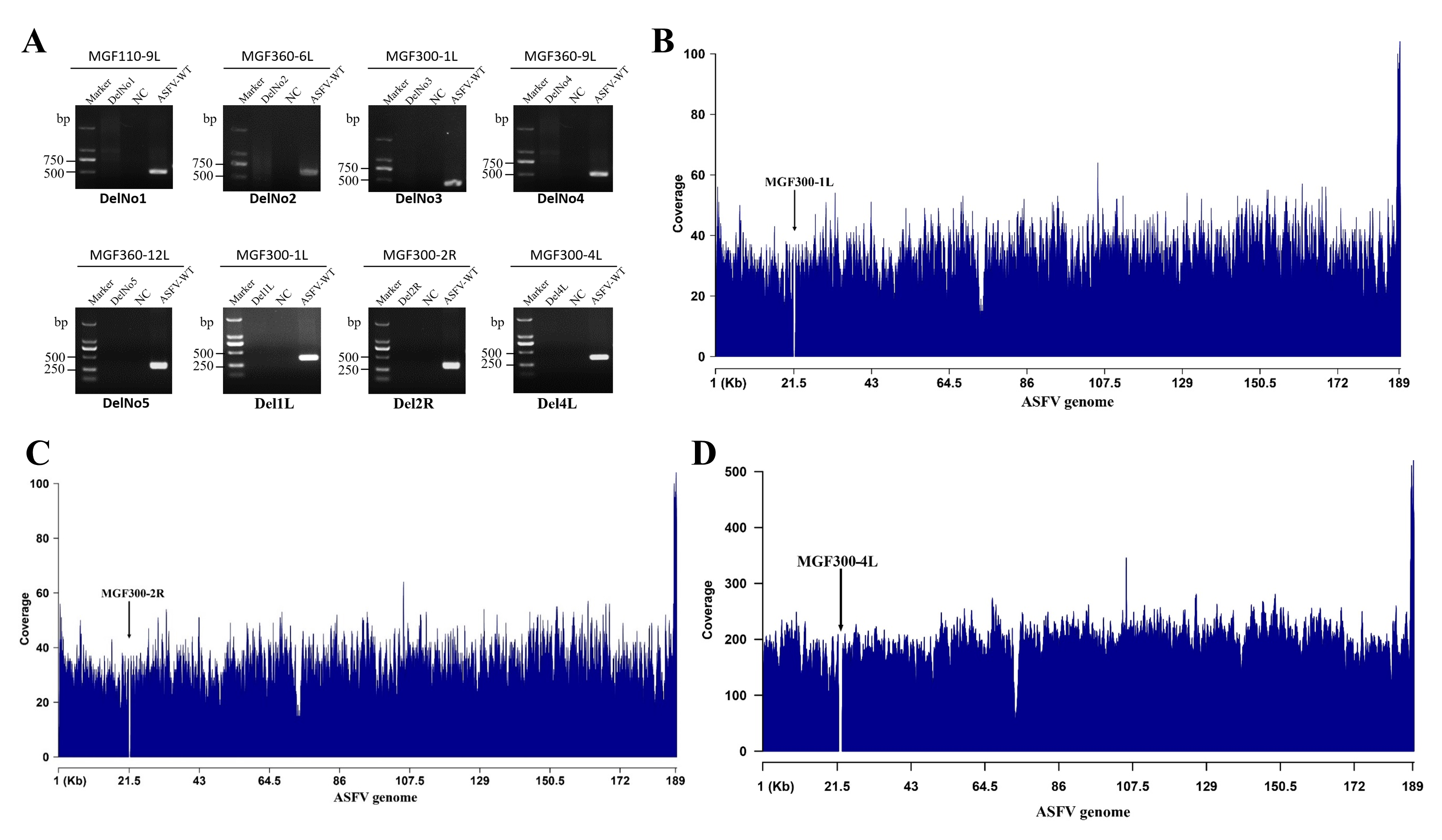

Supplement: S1 Fig — (A) The PCR results of amplifying the genomic segment containing the targeted genes. NC: negative control. Complete genome coverage plot using the NGS reads of the recombinant viruses Del1L (B), Del2R (C), and Del4L (D) mapped against the ASFV HLJ/18 genome. The read coverage values of Del1L, Del2R, and Del 4L were calculated using Samtools and plotted using the Rstudio software (v3.6.1). (TIF) [file ppat.1011580.s001.tif]

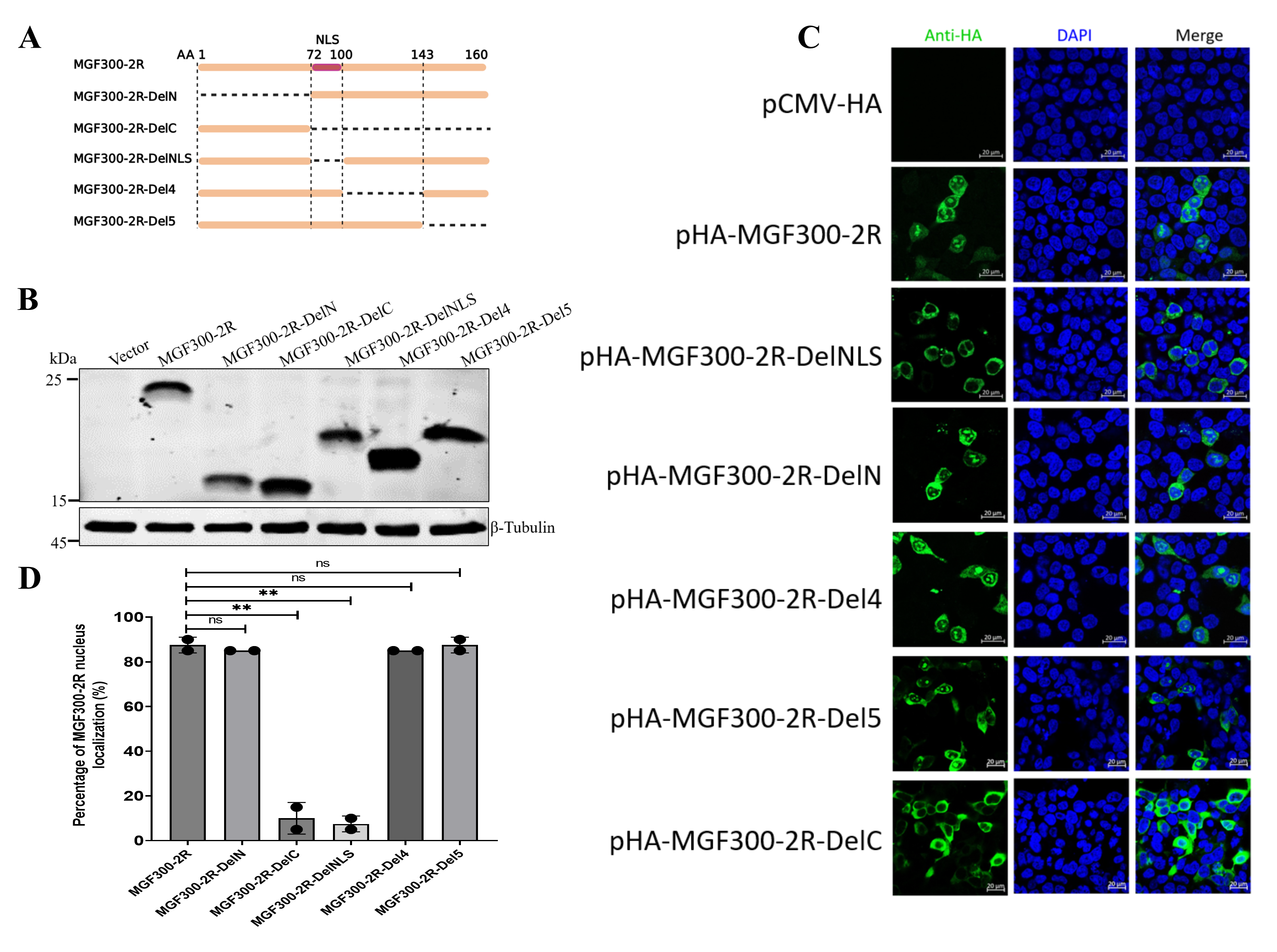

Supplement: S2 Fig — (A) Schematic representation of truncation mutants of MGF300-2R. Dashed lines indicate the range of deleted amino acid relative to the MGF300-2R protein. (B) HEK293T cells were transfected with the mutant-expressing plasmids for 24 h, and the expression of the indicated mutants was detected by western blotting. (C) HEK293T cells were transfected with the mutant-expressing plasmids for 24 h and the subcellular localization of the indicated mutants was examined by confocal microscopy. Bars, 20 μm. (D) Twenty cells were counted in randomly selected view fields two times, and the MGF300-2R nuclear localization ratio was evaluated. (TIF) [file ppat.1011580.s002.tif]

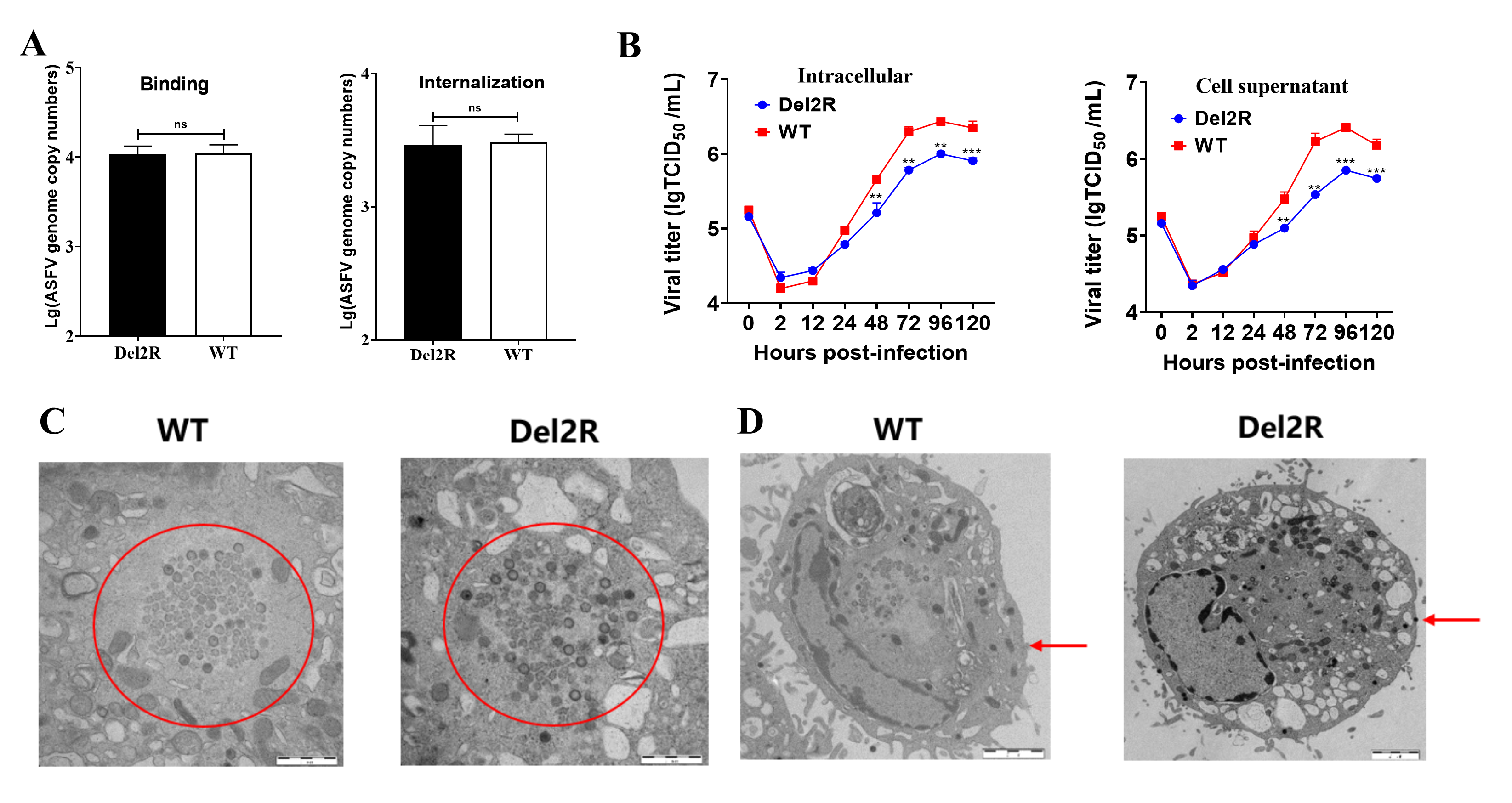

Supplement: S3 Fig — PAMs were infected with Del2R or ASFV-WT (MOI = 5). Binding, internalization (A), and release of virions (B) were analyzed. (C) Morphology of virus factories (red circles) in the Del2R or ASFV-WT infected PAMs (MOI = 5) at 24 hpi. Bars, 1 μm. (D) PAMs were infected with Del2R or ASFV-WT (MOI = 5) and fixed with 2% glutaraldehyde at 24 hpi. The viral budding process (arrowheads) was analyzed by transmission electron microscopy. Bars, 2 μm. (TIF) [file ppat.1011580.s003.tif]

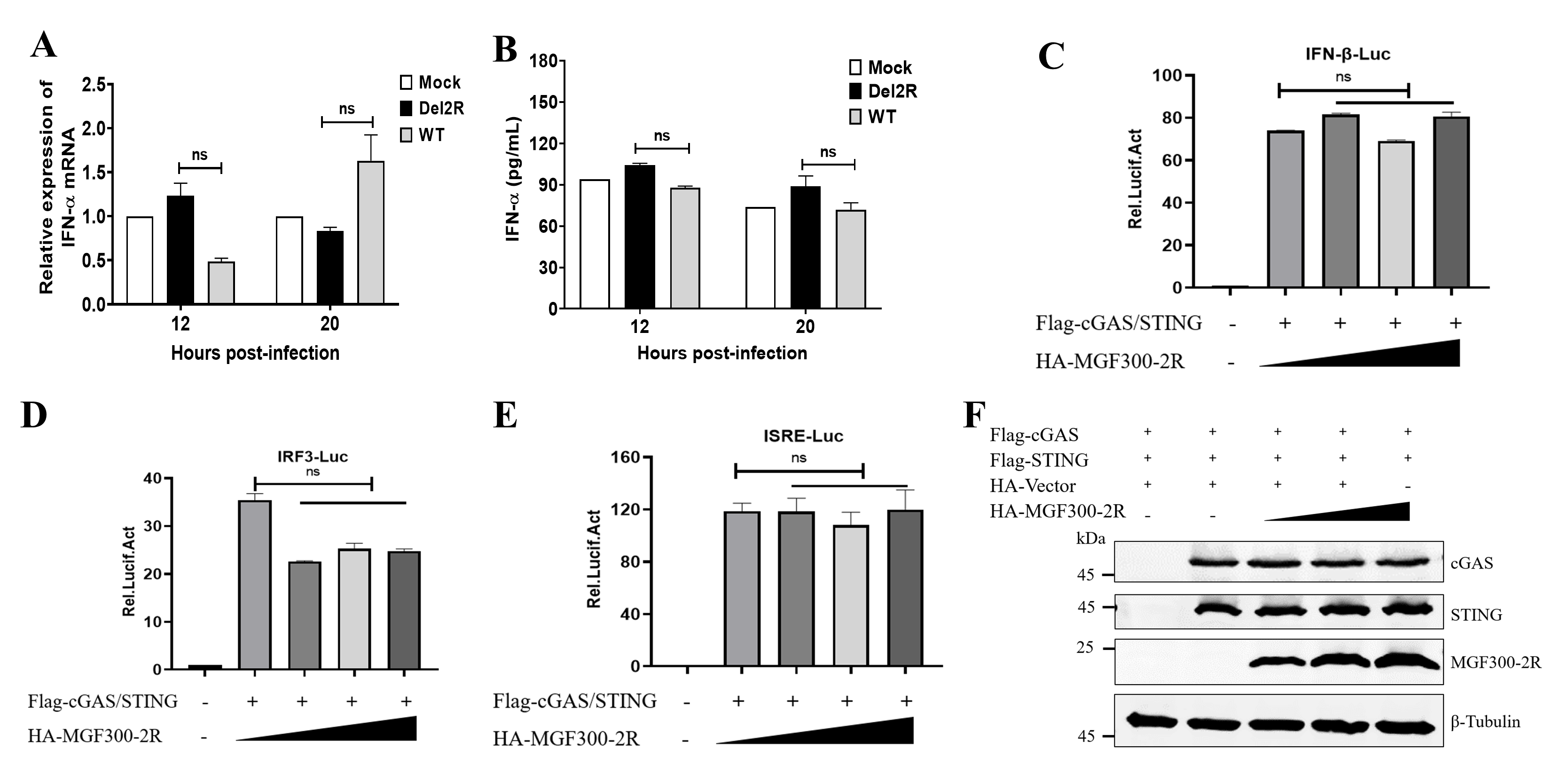

Supplement: S4 Fig — (A and B) PAMs were either mock infected or infected with Del2R or ASFV-WT (MOI = 5). At 12 and 20 hpi, the mRNA levels of IFN-α (A) in the cell lysates were determined by RT-qPCR and the production of IFN-α (B) in the cell culture supernatants was detected by ELISA kits. (C to E) HEK293T cells were cotransfected with increasing amounts of the expressing plasmid pHA-MGF300-2R (0.5, 1, and 1.5 μg) along with IFN-β (C), IRF3 (D), or ISRE (E) promoter reporter and the luciferase activities were measured. (F) The protein expression of MGF300-2R, cGAS, and STING was analyzed by western blotting. (TIF) [file ppat.1011580.s004.tif]

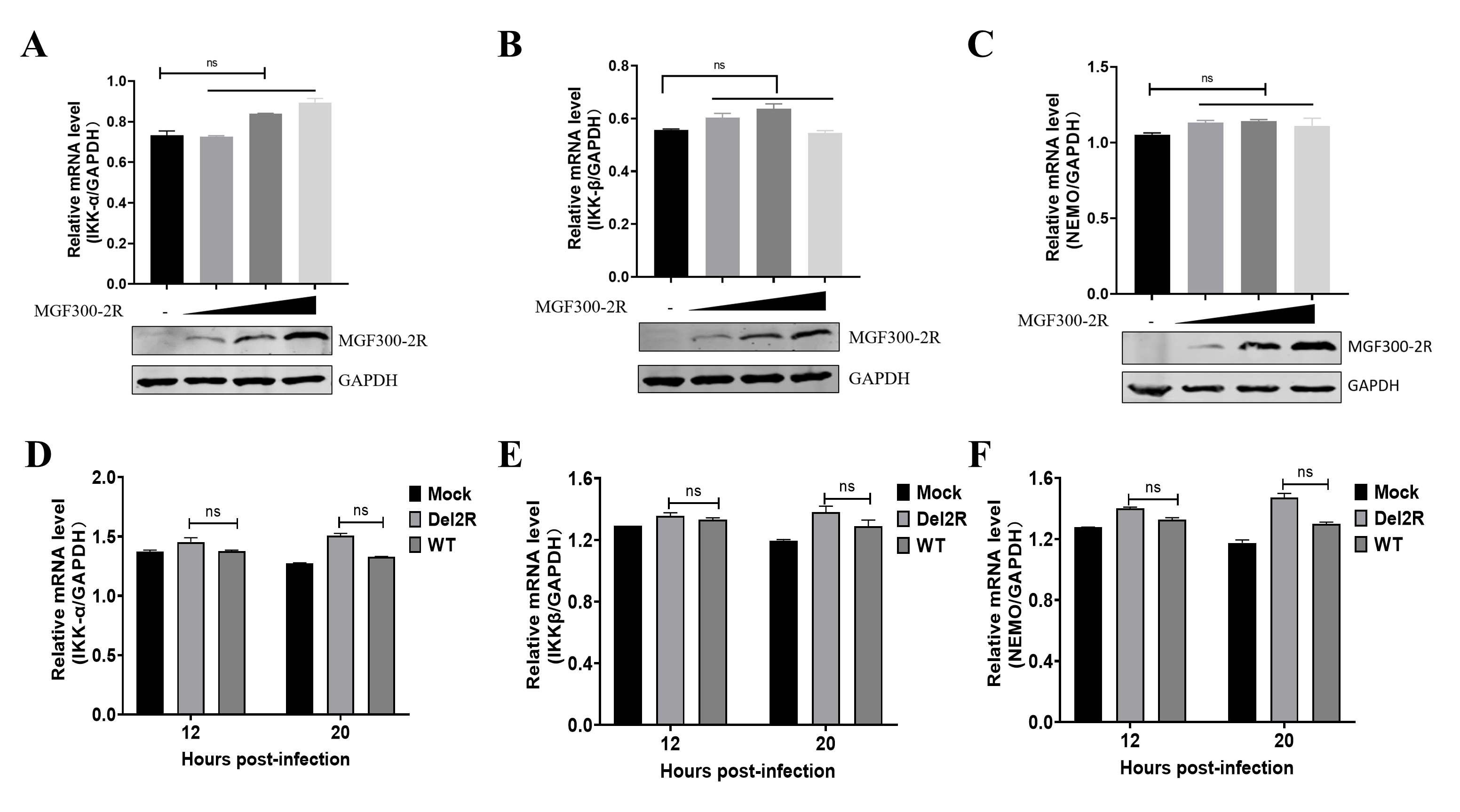

Supplement: S5 Fig — HEK293T cells were transfected with increasing amounts of the expressing plasmid pHA-MGF300-2R (0.5, 1, and 1.5 μg), and the mRNA levels of IKKα (A), IKKβ (B), and NEMO (C) were determined by RT-qPCR. PAMs were either mock infected or infected with Del2R or ASFV-WT (MOI = 5). At 12 and 20 hpi, the mRNA levels of IKKα (D), IKKβ (E), and NEMO (F) in the cell lysates were determined by RT-qPCR. (TIF) [file ppat.1011580.s005.tif]

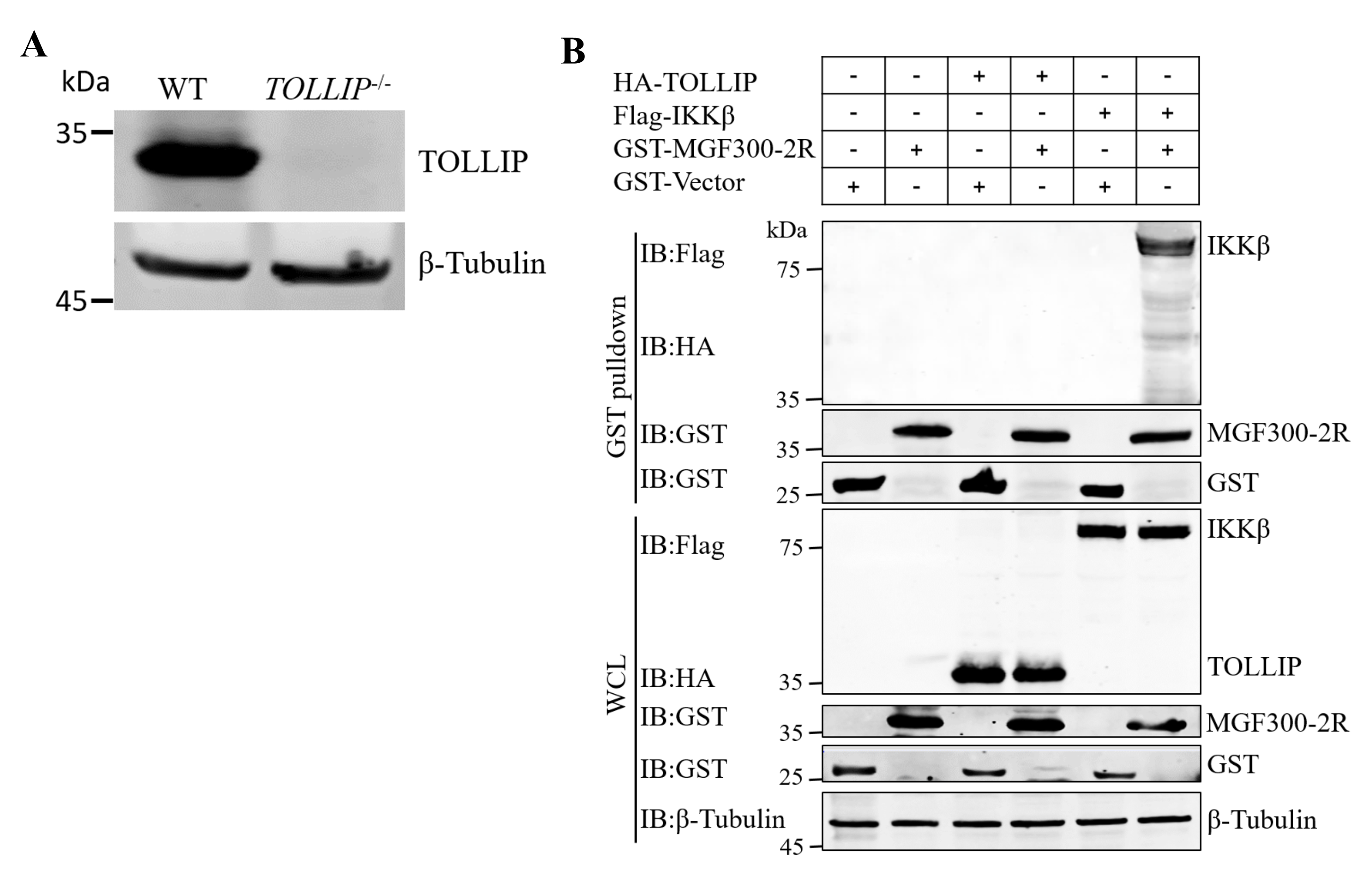

Supplement: S6 Fig — (A) TOLLIP expression was determined by western blotting with antibodies against TOLLIP and β-tubulin. (B) HEK293T cells were transfected with the expressing plasmid pHA-TOLLIP or pFlag-IKKβ for 36 h and lysed with NP-40 buffer. The purified GST or GST-MGF300-2R protein was used to pull down the TOLLIP or IKKβ in the lysates and analyzed by western blotting with the indicated antibodies. (TIF) [file ppat.1011580.s006.tif]
